# The chromosome-scale reference genome of mirid bugs (*Adelphocoris* *suturalis*) genome provides insights into omnivory, insecticide resistance, and survival adaptation

**DOI:** 10.1186/s12915-023-01666-3

**Published:** 2023-09-19

**Authors:** Zhongping Xu, Guanying Wang, Jing Luo, Mingju Zhu, Lisong Hu, Sijia Liang, Bo Li, Xingxing Huang, Ying Wang, Guangyu Zhang, Can Zhang, Yi Zhou, Daojun Yuan, Taiyu Chen, Lizhen Chen, Weihua Ma, Wei Gao, Keith Lindsey, Xianlong Zhang, Fang Ding, Shuangxia Jin

**Affiliations:** 1 National Key Laboratory of Crop Genetic Improvement, Hubei Hongshan Laboratory, Huazhong Agricultural University, Wuhan, Hubei China; 2 State Key Laboratory of Biocatalysis and Enzyme Engineering, School of Life Sciences, Hubei University, Wuhan, Hubei China; 3 Spice and Beverage Research Institute, Chinese Academy of Tropical Agricultural Sciences, Wanning, Hainan China; 4 Academy of Industry Innovation and Development, Huanghuai University, Zhumadian, Henan China; 5 Xinjiang Key Laboratory of Crop Biotechnology, Institute of Nuclear and Biological Technology, Xinjiang Academy of Agricultural Sciences, Wulumuqi, Xinjiang China; 6 Hubei Insect Resources Utilization and Sustainable Pest Management Key Laboratory, Huazhong Agricultural University, Wuhan, Hubei China; 7 State Key Laboratory of Cotton Biology, Henan Key Laboratory of Plant Stress Biology, School of Life Science, Henan University, Kaifeng, Henan China; 8 Department of Biosciences, Durham University, Durham, DH1 3LE UK; 9 Hubei Key Laboratory of Plant Pathology, College of Plant Science and Technology, Huazhong Agricultural University, Wuhan, Hubei China

**Keywords:** *Adelphocoris suturalis*, Chromosome-scale reference genome, Candidate effectors, Omnivory, Detoxification

## Abstract

**Background:**

*Adelphocoris suturalis* (Hemiptera: Miridae) is a notorious agricultural pest, which causes serious economic losses to a diverse range of agricultural crops around the world. The poor understanding of its genomic characteristics has seriously hindered the establishment of sustainable and environment-friendly agricultural pest management through biotechnology and biological insecticides.

**Results:**

Here, we report a chromosome-level assembled genome of *A. suturalis* by integrating Illumina short reads, PacBio, 10x Chromium, and Hi-C mapping technologies. The resulting 1.29 Gb assembly contains twelve chromosomal pseudomolecules with an N50 of 1.4 and 120.6 Mb for the contigs and scaffolds, respectively, and carries 20,010 protein-coding genes. The considerable size of the *A. suturalis* genome is predominantly attributed to a high amount of retrotransposons, especially long interspersed nuclear elements (LINEs). Transcriptomic and phylogenetic analyses suggest that *A. suturalis*-specific candidate effectors, and expansion and expression of gene families associated with omnivory, insecticide resistance and reproductive characteristics, such as digestion, detoxification, chemosensory receptors and long-distance migration likely contribute to its strong environmental adaptability and ability to damage crops. Additionally, 19 highly credible effector candidates were identified and transiently overexpressed in *Nicotiana benthamiana* for functional assays and potential targeting for insect resistance genetic engineering.

**Conclusions:**

The high-quality genome of *A. suturalis* provides an important genomic landscape for further investigations into the mechanisms of omnivory, insecticide resistance and survival adaptation, and for the development of integrated management strategies.

**Supplementary Information:**

The online version contains supplementary material available at 10.1186/s12915-023-01666-3.

## Background

The Miridae of Hemiptera is a hyperdiverse family that includes more than 11,020 species from more than 1200 genera [1, 2]. *Adelphocoris suturalis*, one of the most notorious mirid bugs, has attracted much attention because it causes damage on more than 270 plant species and cause significant economic losses worldwide [3, 4], especially in *Bacillus thuringiensis* (Bt) transgenic cotton growing regions [5, 6] because *A. suturalis* is not affected by the pesticide spectrum of Bt toxin. Therefore, both nymphs and adults induce the stunting of cotton plants and the abscission of flower buds even cotton bolls (Additional file 1: Figure S1), resulting in serious yield and quality losses [7]. *A. suturalis* is emerging as a super pest insect worldwide due to the following characteristics: (1) omnivory: enabling it to feed upon plants and other arthropods, such as *Aphis gossypii*, during larval and adult development; (2) strong long-distance migratory capabilities, allowing it to effectively avoid natural enemies and pesticides; (3) a wide range of detoxification mechanisms, facilitating quick establishment of resistance to pesticides. In addition to directly sucking plant sap and directly damaging crops, *A. suturalis* also acts as an important vector for transmitting plant viruses that cause significant yield losses [8, 9].

Currently, the sole effective management strategy for *A. suturalis* is through the application of insecticides, such as organophosphates and pyrethroids [10]. However, the extremely rapid population expansion and prolonged dependence on insecticide use has led to the development of resistance among the target populations [10], in addition to serious environmental and safety issues. Genetic engineering controls, such as RNA interference (RNAi) technology, have been used in field pest management measures after proving its efficacy in impairing the resistance of cotton bollworm to gossypol [11]. Subsequently, our group applied an RNAi strategy to suppress *A. suturalis*’ fatty acyl-CoA reductases (*FARs*), an enzyme essential for female fertility, leading to significantly suppressed ovarian development of *A. suturalis* and resistant to damage caused by plant bug infestation in cotton [7]. A similar RNAi strategy from our group also showed impaired growth of *Apolygus lucorum* when feeding on transgenic cotton plants expressing dsRNA for the *LIM* gene, required for muscle cell growth and differentiation [12]. However, non-lethal effects, comparable to those observed with Bt toxin for cotton bollworm, were detected in these RNAi insect control studies due to poor understanding of *A. suturalis*, which makes it unreachable to select suitable target for RNAi. The limited availability of suitable target genes for RNAi severely hinders the use of RNAi transgenic crops for large-scale control of mirid bugs in the field. Therefore, a better understanding of the mechanisms controlling omnivory, detoxification, reproduction, sex pheromones and migration will help identify essential target genes to develop of novel biological control strategies [13].

Here, we report high-quality genomes of *A. suturalis* which were assembled utilizing Illumina short reads, PacBio long reads, 10x Chromium and high-throughput chromosome conformation capture (Hi-C) technology. Following these high-quality genomes, we performed extensive genome-wide comparative studies to reveal complex genomic characteristics and evolution, as well as assessed changes in gene families relevant to feeding and survival strategies. Additionally, a total of 19 high-credibility effector candidates were identified and transiently overexpressed in plants to assay their functions. The availability of the *A. suturalis* genome not only provides novel insights into the underlying mechanisms of its global invasiveness but also presents new opportunities in plant protection against mirid bug pests.

## Results

### Assembly of high-quality *A. suturalis* genomes with comprehensive strategy

**Fig. 1:**
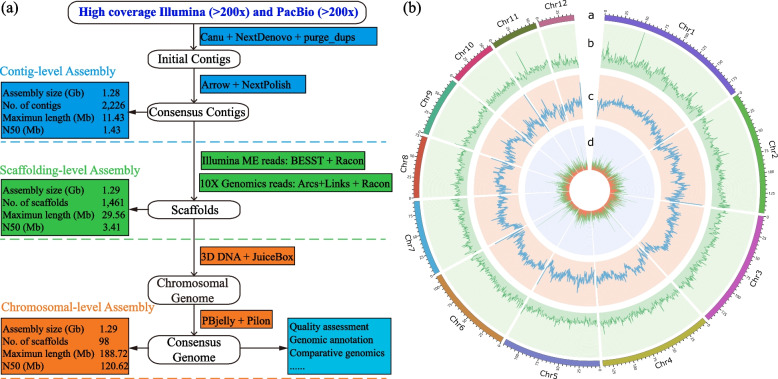
Overview of the *A. suturalis* genome assembly and features. **a** Genome assembly pipeline used for the *A. suturalis*. **b** Distribution of *A. suturalis* genomic features. Track “a” represents the assembled chromosomes. Tracks “b–d” represents the distribution of GC density, repeat density, and gene density, respectively, with densities calculated in 500-kb windows

To mitigate the heterozygosity of the sequenced *A. suturalis* genome, a colony established from a single female was collected from Huazhong Agricultural University in Wuhan, Hubei for genome sequencing. The genome size was estimated to be approximately 1.29 Gb with high heterozygosity (1.7%) using a k-mer genome survey analysis approach (Additional file 1: Figure S2). Given the complex genomic characteristics, firstly, a total of 610 Gb 150 Illumina pair-reads and 128 Gb 150 Illumina mate-reads with different insert sizes (Additional file 2: Table S1), along with 156 Gb PacBio RS II and 326 Gb PacBio Sequel II long reads (Additional file 2: Table S2) were obtained to perform contigs assembly (Fig. 1a). The PacBio long reads were corrected through Canu and subsequently inputted into NextDenovo for initial contig assembly, followed by purging the haplotigs and generating a consensus with Arrow and NextPolish using both PacBio and Illumina reads, which ultimately generated an *A. suturalis* genome of 1.28 Gb with a contig N50 of 1.4 Mb. Subsequently, using BESST and LINKS with Illumina mate-reads and 10x Chromium Linked-reads (~ 476 Gb, Additional file 2: Table S3), we ordered and oriented linked contigs into longer scaffolds, which resulted in an assembly consisting of 1461 scaffolds with an N50 of 3.4 Mb. Finally, using 100x coverage reads from Hi-C libraries (Additional file 2: Table S4), a total of 1.25 Gb (97.2%) assembled sequences were scaffolded on 12 chromosomes (Fig. 1b). After postprocessing by gap filling and polishing, the final assembly has a size of 1.29 Gb and super-scaffold N50 length of 120.1 Mb (Table 1 and Additional file 2: Table S5), indicating that this assembly reaches a reference grade for quality.

**Table 1:** Summary of the genome assemblies for *Adelphocoris suturalis*

	*Adelphocoris suturalis*
Sequencing platform	Illumina + PacBio RS II + PacBio Sequel II
Genome sequencing depth (x)	260
Genome sequencing depth Hi-C	106
Estimated genome size (Gb)	1.29
Assembly	
Number of scaffolds	98
Sequenced genome size (Gb)	1.29
Contig N50 (Mb)	1.43
Scaffold N50 (Mb)	120.63
Longest scaffold (Mb)	188.72
GC content (%)	38.38
Repetitive sequences (%)	72.76
Annotated protein-coding genes	20,010
BUSCO completeness of assembly (%)	95.3
BUSCO completeness of annotation (%)	86.4

Assembly completeness was then evaluated by aligning the Illumina short reads against the *A. suturalis* genome assemblies and resulting in a mapping rate of 89.73% (Additional file 2: Table S6). Furthermore, Benchmarking Universal Single-Copy Orthologs (BUSCO) analysis with insecta_odb10 revealed that 95.3% of genes were found in the genome, including 1303 (93.3%) single-copy, 8 (0.6%) fragmented and 56 (4.1%) missing BUSCO genes (Additional file 2: Table S7 and Figure S3). In addition, the heatmap of the Hi-C interaction matrix for each chromosome also showed no signs of misorientations or other large-scale structural errors (Additional file 1: Figure S4). These data support that assemblies are of high quality and virtually complete.

A comprehensive gene model prediction integrated with homology-based prediction, RNA-sequencing-assisted prediction and ab initio prediction identified a total of 20,010 protein-coding genes with 86.4% complete BUSCO genes (Additional file 2: Table S8 and Figure S3), indicating that nearly all potential genes are identified in this assembled genome. Of these, more than 94.3% genes are functionally annotated following searches of NR, GO, KEGG, SwissProt and TrEMBL databases. In addition, 2149 tRNAs and 1113 rRNAs were also identified through structural characteristics and alignment to the database.

The highly continuous genome enables a comprehensive study of transposable elements (TEs). 72.76% of the *A. suturalis* genome comprised repetitive sequences. Among these repeats, 69.51% are classified as interspersed repeats and the major types of TEs are long terminal repeat (LTR; 192 Mb) at 14.9%, 33.2% LINE (427 Mb) and 19.5% DNA (250 Mb) elements (Additional file 2: Table S9). Similar to most other genomes, the predominant type of LTR retrotransposons are LTR/Gypsy (10.31%) and LTR/Copia (2.55%) retro-elements. Interestingly, we found that DNA and LINE transposons showed a recent activity burst (Additional file 1: Figure S5), similar to those reported in *A. lucorum* [14] and other insect genome [15]. Moreover, these TEs exhibit an apparently random distribution on the chromosomes, though with an inverse correlation with gene density (permutation test, *p*-value < 0.05; Fig. 1b and Additional file 1: Figure S6).

### Genome comparisons and genome-based phylogeny

**Fig. 2:**
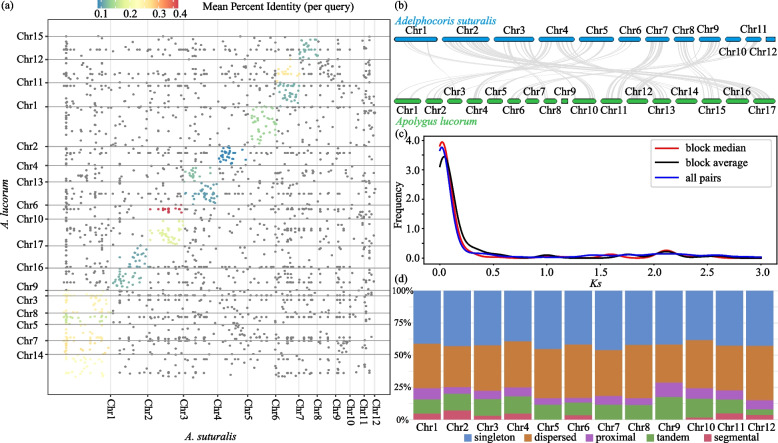
Comparative genome and gene duplication in *A. suturalis*. **a** Dotplots of inter-species syntenic blocks of *A. suturalis* and *A. lucorum* using genome data. Each dot indicates a syntenic gene pair detected by minimap2. **b** Syntenic comparison between *A. suturalis* and *A. lucorum* using transcriptome data. The syntenic gene pairs were detected by jcvi. **c** Distribution of synonymous mutation rate (*Ks*) values for paralogous gene pairs in *A. suturalis*, defined by reciprocal best blast hit. **d** Classification of gene duplicates origin in *A. lucorum* genome. The origins of gene duplicates were classified into five types: whole-genome/segmental duplication, tandem duplication, proximal duplication, dispersed duplication, and singleton

**Fig. 3:**
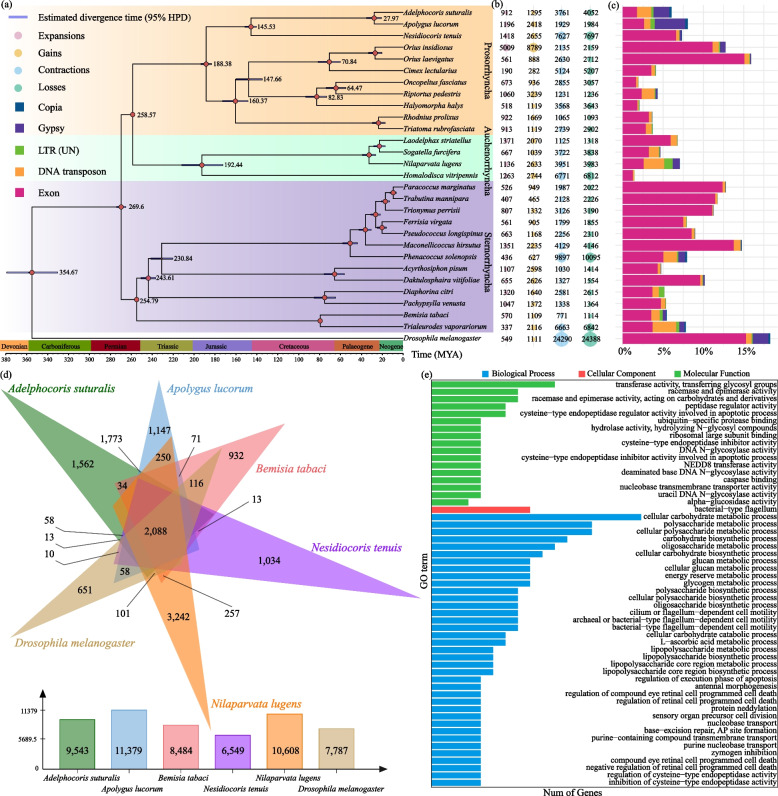
Analyses of gene family evolution of *A. suturalis*.** a** Inferred maximum likelihood phylogenetic tree by for *A. suturalis* and other sequenced species in Hemiptera. Divergence timings and the supported bootstrap values were labeled on the tree at the internodes with 95% highest posterior density (HPD). Mya million years ago. **b** The number indicate the expansions, gains, contractions, and losses of gene families of the corresponding species. **c** The percentage of transposons and exons in genome sequence of the corresponding species. Intact LTR retrotransposons and DNA transposons were used to stats. **d** Venn diagram of shared orthologous genes’ families by *A. suturalis* and five other species genomes. **e** GO enrichment results of 9509 expanded gene families of *A. suturalis* (biological process category)

*A. suturalis* and* A. lucorum* belong to different genera of the Miridae family, but both major sucking pests endangering crop productivity. However, the synteny analysis of genomes and genes showed low similarities between them (Fig. 2a, b), which included 2183 orthologous genes that account for 10.9 and 11.3% of the total number of genes in *A. suturalis* and* A. lucorum*, respectively. Gene ontology (GO) analyses of these orthologous genes revealed significant enriched GO terms involved in larval somatic muscle development, detoxification, sensory perception of smell and sensory perception of chemical stimulus (Fisher’s exact test, adjusted *p*-value < 0.05; Additional file 2: Table S10). Moreover, the Kyoto Encyclopedia of Genes and Genomes (KEGG) enriched pathways related to terpenoid backbone biosynthesis, glycerolipid metabolism, and carbon metabolism, as well as the metabolism of amino acids, such as cysteine and methionine (Additional file 2: Table S11). In addition to the differences in coding genes, comparison with *A. lucorum*, *A. suturalis* genome contains a high proportion of DNA transposons (nearly three times) and low proportion of LTR transposons (about 1.5 times) (Fig. 3c).

Genomic synteny analysis based on the self-comparison of *A. suturalis* protein-coding genes shows 144 synteny blocks including 1751 genes (~ 8.75% of all genes). Distribution of synonymous distances (*Ks*) shows that 1198 (68.42%) paralogue pairs had a *Ks* value smaller than 0.1, suggesting that most gene duplications possibly occurred in a recent period (Fig. 2c). We also analyzed the duplicate gene origins in *A. suturalis*. The results suggest that dispersed duplication is the predominant type (35.77%, 7,157) as compared to the whole-genome duplication (WGD)/segmental duplication (5.03%, 1006), tandem duplication (10.69%, 2140), and proximal duplication (6.22%, 1244) (Fig. 2d), similar to the *A. lucorum* genome [14] and indicating that duplicated genes in *A. suturalis* are mostly derived from small local-scale gene duplications, rather than whole-genome duplication. Moreover, chromosome 9 contains the highest proportion of proximal and tandem duplication compared to other chromosomes (Fig. 2d). KEGG analyses of these tandem genes revealed significant enrichment in insect hormone biosynthesis, folate biosynthesis and galactose metabolism (Fisher’s exact test, adjusted *p*-value < 0.05).

Then, *A. suturalis* protein-coding genes were compared with all those available in the Hemiptera and *Drosophila melanogaster* as the outgroup to identify orthologous groups. The phylogeny, based on 203 single-copy orthologous genes, using both coalescent and concatenation analyses showed that *A. suturalis* is a sister taxon to *A. lucorum* after their divergence from *Nesidiocoris tenuis* (Fig. 3a). Based on the high-confidence phylogenetic tree and calibration points, the divergence time between *A. suturalis* and *A. lucorum* is estimated to be 27.97 million years ago (MYA; 4.3–17.8 MYA), well after the divergence from *N. tenuis* ~ 145.53 MYA (4.3–17.8 MYA) (Fig. 3a).

To reveal the genomic basis of the distinctive phenotypes of *A. suturalis*, we investigated the evolution of gene families by characterizing unique and shared gene families among 29 insects that have been used for phylogenetic tree reconstruction. Through comparing gene families, we classified 12,321 genes of *A. suturalis* into 9543 gene families. Meanwhile, gene family expansion and contraction analysis classified 912, 1295, 3761 and 4052 gene families as expanded, genes gained, contracted and genes lost, respectively (Fig. 3b), among which 50 were identified as rapidly evolving families (10 expanded and 40 contracted). Functional annotation of these 10 rapidly expanded gene families reveals that they are involved in glutathione metabolism. Meanwhile, the 40 rapidly contracted gene families are annotated as being related to the fatty acid metabolism and amino acid metabolism, including glycine, serine, arginine, proline, threonine, cysteine, and methionine. Further comparison among the species spanning the entire phylogenetic tree (*A. suturalis*, *A. lucorum*, *N. tenuis*, *Bemisia tabaci*, *Nilaparvata lugens* and *D. melanogaster*; Fig. 3a) reveals 2088 gene families distributed among all six genomes; 1562 gene families were unique to *A. suturalis* (Fig. 3d). KEGG enrichment analysis of these 1562 gene families revealed significant pathway that include “ABC transporters,” “biosynthesis of amino acids,” “pentose phosphate pathway,” “glycerophospholipid metabolism,” and “insect hormone biosynthesis” (Fig. 3e and Additional file 2: Table S12).

### Expansion and widespread expression of detoxification enzyme gene family in *A. suturalis*

**Fig. 4:**
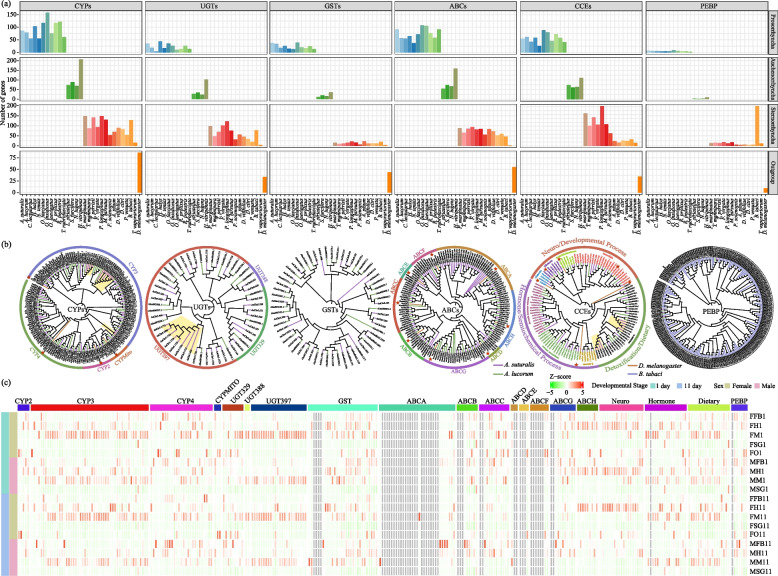
Evolution of detoxification enzymes and insecticide resistance genes in *A. suturalis*. **a** Gene number comparison of detoxification enzymes and insecticide resistance genes encoding cytochrome P450 (CYP), UDP-glucuronosyltransferase (UGT), glutathione S-transferase (GST), ABC transporter (ABC), carboxylesterase (CCE), and phosphatidylethanolamine-binding protein (PEBP) between *A. suturalis* and other species in Hemiptera. **b** Phylogenetic tree of CYPs, UGTs, GSTs, ABCs, CCEs, and PEBPs in *A. suturalis* and other species. The *A. suturalis*-specific expanded clades are emphasized with yellow shadow. Red asterisks indicate reference sequences from *Drosophila melanogaster* (Dm) genome database (http://flybase.org/) that are used to classify and annotate these gene families. Different clades are labeled in different colors. **c** Expression profiles of *A. suturalis* CYPs, UGTs, GSTs, ABCs, CCEs, and PEBPs genes from individuals at different tissues and developmental stages. The color intensity changes with the relative expression from low (green) to high (red). The color bar above the heatmap indicates different gene families or subfamilies. F, female; M, male; FB, fat body; H, head; M, midgut; O, ovary; SG, salivary gland; 1, 1 day; 11, 11 day

The enhancement of detoxification metabolism is the major mechanism of insect resistance to insecticides. The sequencing of the genome of *A. suturalis*, a highly polyphagous agricultural pest, gives us the opportunity to identify the detoxification enzymes related to resistance and study the biochemical mechanism of metabolic resistance to insecticides and plant toxic secondary metabolite such as terpenes, alkaloids and flavonoids. Several enzyme families implicated in detoxification were identified in the *A. suturalis* genome, including cytochrome P450s (*CYPs*), UDP-glucuronosyltransferases (*UGTs*), glutathione S-transferases (*GSTs*), ATP-binding cassette transporters (*ABCs*), carboxylesterases (*CCEs*), and phosphatidylethanolamine-binding protein (*PEBP*) (Additional file 1: Note 1, Fig. 4 and Additional file 2: Table S13).

A total of 86 P450s containing five *CYP2s*, 51 *CYP3s*, 27 *CYP4s*, and three *CYPMitos* have been identified in the genome of *A. suturalis*, among which CYP3 shows an expansion relative to the most close species *A. lucorum* (Fig. 4a). Members of the CYP3 clade have been implicated in the oxidative detoxification of plant secondary metabolites and synthetic insecticides [16, 17]. The phylogenetic tree of *CYPs* exhibited three *A. suturalis*-specific branches in the CYP3 clade (Fig. 4b). What is more, most *CYP* genes show tandem duplications and the largest CYP cluster was located on Chr3 and belong to CYP3 clade (Additional file 1: Figure S7). The species-specific expansion and tandem duplications of *CYPs* may offer *A. suturalis* stronger detoxification ability and insecticide resistance. Moreover, the *CYP3* gene families tended to be expressed in the midgut of both sexually immature (1 day old) and sexually mature (11 days old) females and males (Fig. 4c). Meanwhile, we found that all these *CYP* genes are widely expressed at major developmental stages and in all tissues, except for relatively low expression in the salivary gland, suggesting the importance of *CYP* genes in all life stages for *A. suturalis* to detoxify plant xenobiotics.

UGTs are a multifunctional superfamily of enzymes that widely exist in animals, plants, bacteria and viruses. Total 35 putative *UGTs* were manually annotated in the *A. suturalis* genome, which is greater than in most other species of Prosorrhyncha (Hemiptera suborder, with only 19 in *A. lucorum*; Fig. 4a). Moreover, approximately 91% of *A. suturalis UGTs* are arranged in a tandem manner and 13 of them are concentrated in one cluster on Chr5 (Additional file 1: Figure S8). Phylogenetic analysis shows that the largest *UGT* family observed in *A. suturalis*, UGT397, consists of 22 genes and exhibits a significant *A. suturalis*-specific expansion compared with *A. lucorum* (Fig. 4b). All *UGT* genes are widely expressed across different tissues and developmental stages in *A. suturalis*, and especially strongly expressed in the midgut (Fig. 4c).

GSTs mainly catalyzes the covalent conjugation of glutathione with toxic electrophilic and hydrophobic substrates, so as to participate in a variety of detoxification metabolic processes in insects [18]. Additionally, 30 *GSTs* were identified in *A. suturalis* (Fig. 4a). Phylogenetic and expression profile analysis showed that most *GSTs* genes were expressed in various tissues, and especially high expression in the midgut is in line with their detoxification function (Fig. 4b, c).

ABCs form the largest family of transmembrane proteins and bind and hydrolyze ATP and use the energy of this reaction to drive the transport of various substrates across membranes, with role in detoxification, defense and protection to various tissues and organs [19]. A total of 90 *ABC* genes that grouped into 8 families were found in the genome of *A. suturalis*, more than in other Hemiptera species (Fig. 4a, b). The ABCA subfamily, which is involved in lipid metabolism and promotes the development of wings and elytra or provides energy for flight [20, 21], was the largest subfamily in *A. suturalis*. The expression profiles showed that 51% genes were widely expressed in sampled tissues of *A. suturalis* (Fig. 4c).

CCEs provide the means for insects to resist exogenous pesticides. A total of 55 *CCE* genes were detected in *A. suturalis* genome (Fig. 4a). The constructed phylogeny tree shows that all the *CCEs* fall into three main phylogenetic classes of dietary/detoxification, hormone/semiochemical processing and neuro/developmental functions (Fig. 4b). Among these, 18 *CCE* genes belong to the α-esterase clade and are largely arranged in a tandem manner on Chr5 (Additional file 1: Figure S9). Eighteen *CCE* genes were involved in hormone/semiochemical processes with 8 juvenile hormone esterase, 3 β-esterases, and 7 integument esterases. The remaining 19 *CCE* genes are in the neuro/developmental processes class, with 2 acetylcholinesterase, 3 gliotactins, 12 neuroligins, and 2 glutactins. The tissue-specific expression pattern of *CCE* genes in *A. suturalis* shows that all *CCE* genes from the neuro/developmental processes class have significantly upregulated expression in the head compared with other tissues. However, other dietary/detoxification and hormone/semiochemical *CCE* genes are highly expressed in the midgut and fat bodies (Fig. 4c). The considerable number of *CCEs* and abundant distribution in fat bodies and the midgut indicates potentially important roles in detoxifying insecticides and thus conferring insecticide resistance in *A. suturalis*.

PEBPs are a highly conserved group of proteins associated various biological processes and have been reported to contribute to insect resistance to pesticides, such as in *B. tabaci* [22]. However, only 7 *PEBPs* in the genome of *A. suturalis* and 5 its related species *A. lucorum* were found (Fig. 4a), which is far lower than the 202 in the *B. tabaci* genome [22]. Phylogenetic tree analysis shows that *PEBPs* in *A. suturalis* and *A. lucorum* are on different evolutionary branches compared to *B. tabaci*, but themselves have the same evolutionary trajectory (Fig. 4b). Moreover, transcriptome analysis showed that *PEBPs* in *A. suturalis* were more highly expressed in the head and fat bodies than in other tissues (Fig. 4c).

In summary, the expansion of detoxification gene families and their wide expression in different tissues at different developmental stages in *A. suturalis* provides a solid genetic basis for its well-known insecticide resistance and its ability to occupy a broad range of host plants with a diversity of defenses.

### High and specific expression of extensive clustered chemosensory receptor genes in the head of *A. suturalis*

**Fig. 5:**
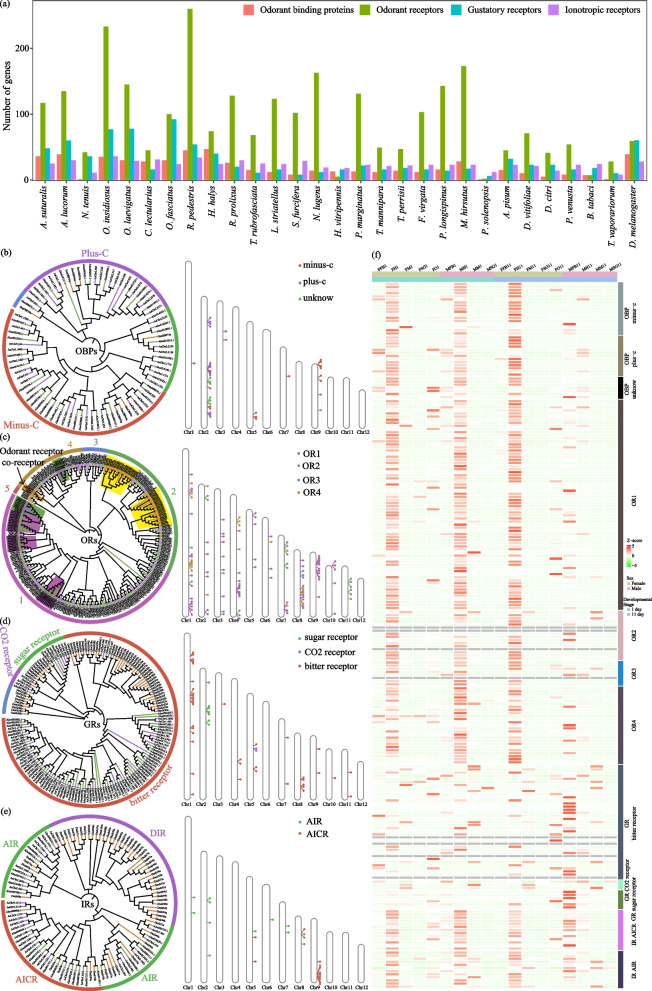
Evolution of chemosensory receptors genes in *A. suturalis*. **a** Gene number comparison of chemosensory receptor genes between *A. suturalis* and other species in Hemiptera. **b**–**e** Maximum likelihood phylogenetic and chromosomes distribution of OBP, OR, GR, and IR genes in *A. suturalis*, *A. lucorum*, and* D. melanogaster* (Dm). The species-specific expanded clades are emphasized with shadow. Different clades are labeled in different colors. **f** Expression profiles of *A. suturalis* OBP, OR, GR, and IR genes from individuals at different tissues and developmental stages. The color intensity changes with the relative expression from low (green) to high (red). The color bar above the heatmap indicates different gene families or subfamilies. F, female; M, male; FB, fat body; H, head; M, midgut; O, ovary; SG, salivary gland; 1, 1 day; 11, 11 day; AIR, antennal ionotropic receptors; AICR, antennal ionotropic co-receptors; DIR, divergent ionotropic receptors

Chemoreception is vital for insects to sense the complex odor substances in the environment, so as to complete in important activities such as finding mates, food, spawning places and avoiding natural enemies. Perception and recognition of odorants involve multiple proteins, such as odorant-binding protein (OBP), odorant receptor (OR), gustatory receptor (GR), and ionotropic receptor (IR). We compared the members from those four gene families in *A. suturalis* with those from other Hemiptera and *D. melanogaster*, which act as a reference to classify genes (Fig. 5a and Additional file 2: Table S14).

We manually annotated 37 *OBP* genes in the *A. suturalis* genome. About 47% of *OBPs* comprise a large expansion of the minus-C subfamily and 13 *OBPs* are identified as members of the plus-C group, contrasting with only one *OBP* gene in this subfamily in *Anoplophora glabripennis* [23], *Tribolium castaneum*, and *Dendroctonus ponderosae* [24] highlighting the difference in evolution between the different orders. Phylogenetic analysis of the OBPs in *A. suturalis* and *A. lucorum* showed similar evolutionary branches, with no species-specific expansion and few examples of gene duplication (Fig. 5b).

In addition to the one highly conserved OR co-receptor Orco (*AsChr6.271* in *A. suturalis* and *Aluc010489.1* in *A. lucorum*), *A. suturalis* has 117 *OR* genes, less than the 135 found in the closely related species *A. lucorum* [14]. Representatives of all five subfamilies and two lineages (1 and 3) of *ORs* were identified in *A. lucorum* and *A. suturalis* specifically, and placed as outgroups to OR groups 2, 4, and 5 in *D. melanogaster* (Fig. 5c). Moreover, three *A. lucorum*-specific and two *A. suturalis*-specific branches are exhibited in the OR phylogenetic tree (Fig. 5c), suggesting these two closely related species may have developed a species-specific chemosensory recognition system, but with subtle differences to recognize and distinguish chemical signals that may be essential to host plant selection. Data for the *D. melanogaster*-specific subfamily 5 suggests that protein sequence identities of ORs are widely divergent and that *OR* genes of insects are poorly conserved, suggesting in turn a basis for recognition of widely different odors to allow the insect to expand their host plant range. Next, a total of 116 *OR* genes were mapped to the 12 chromosomes of *A. suturalis* and distribution analysis shows at least 13 *OR* clusters (Fig. 5b). The largest OR cluster were located on Chr8 and Chr9, each consisting of 10 *OR* genes (Fig. 5a, b).

*A. suturalis* has an extensive suite of 47 *GRs*, including 3 conserved candidate CO_2_ receptors, 6 candidate sugar receptors, and one candidate fructose receptor related to *DmGr43a*. The remaining 38 *GRs* belong to the general category of candidate bitter taste receptors, although some likely are also involved in contact pheromone perception. Interestingly, the CO_2_ receptors lineage consists of three genes compared with only one in *A. lucorum*. Moreover, phylogenetic analysis of the *GR* genes shows that *A. lucorum* and *A. suturalis* have close evolutionary branches that are located on branches different to *D. melanogaster*, suggesting that the *GR* genes have great diversity among different species. Chromosomes distribution analysis shows that 50% *GR* genes are located on Chr1 of *A. suturalis* and exist in two clusters (Fig. 5d).

In addition, 25 *IR* genes were annotated in *A. suturalis*. A phylogenetic analysis conducted with the whole set of IRs revealed the presence of orthologs of conserved antennal ionotropic co-receptors (AICR) and antennal ionotropic receptors (AIR). However, the divergent ionotropic receptors (DIR) that widely exist in *D. melanogaster* are lost in both *A. lucorum* and *A. suturalis* (Fig. 5e). Interestingly, 60% of AICR genes were clustered and located on Chr9 of the *A. suturalis* genome.

Transcriptome analysis showed that 90% of *OBPs*, *ORs*, and *IRs* were highly expressed in the insect head (Fig. 5f), which is the major odorant perception organ in insects. However, some *IRs* are highly expressed in tissues other than the head, notably in fat bodies of 11-day males, reflecting a diversity of IR functions. Indeed, the expansion of *ORs* are remarkable in *A. lucorum* and *A. suturalis* compared to the other Hemiptera species and *D. melanogaster* (Fig. 5a, c), mostly due to recent replication, and may be related to its extensive hosts and host-switching behavior. In contrast, *GRs* exhibit different expression patterns, with most *GRs* expressed in several tissues (Fig. 5f), indicating the diversity of GR functions.

### Expansion of digestive enzyme genes associated with dietary diversity

**Fig. 6:**
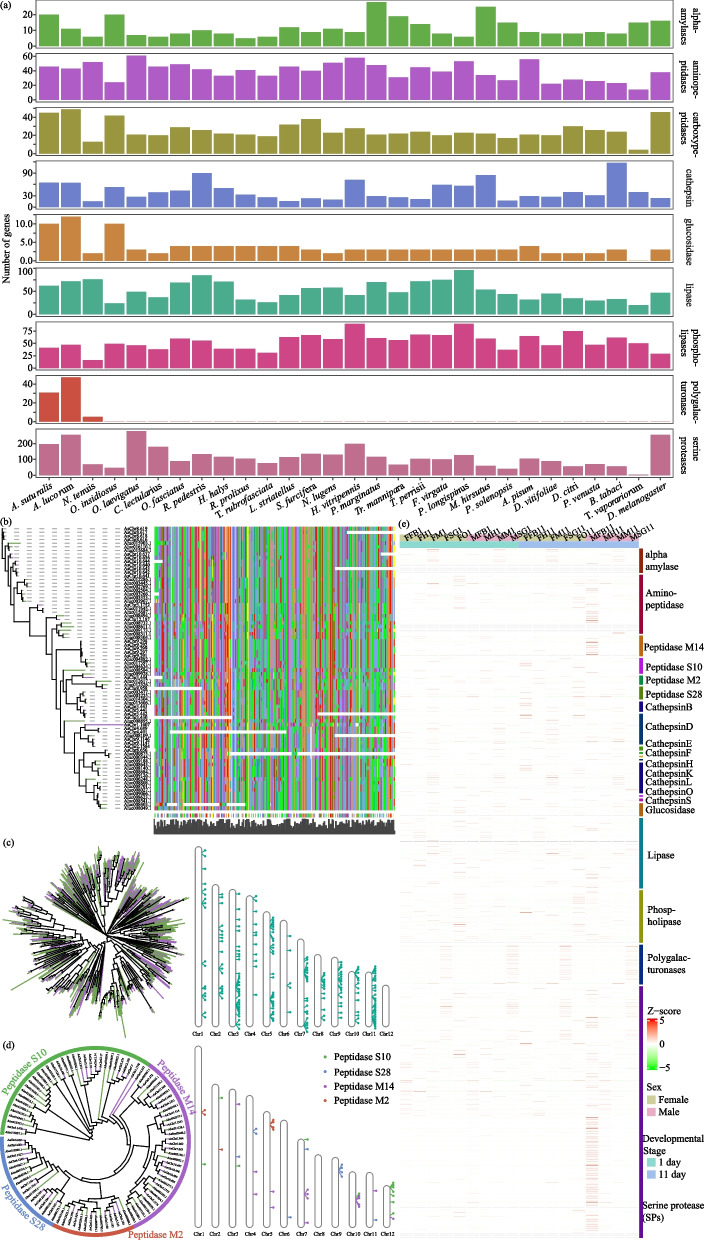
Evolution of digestive enzyme genes in *A. suturalis*.** a** Gene number comparison of digestive enzyme genes between *A. suturalis* and other species in Hemiptera. **b** Phylogenetic relationships and sequence identity of *A. suturalis* with *A. lucorum*. **c**, **d** Maximum likelihood phylogenetic and chromosomes distribution of SPs and carboxypeptidases genes in *A. suturalis* and *A. lucorum*. **e** Expression profiles of *A. suturalis* alpha-amylases, aminopeptidases, carboxypeptidases, cathepsins, glucosidases, lipases, phospholipases, PG, and SP genes from individuals at different tissues and developmental stages. The color intensity changes with the relative expression from low (green) to high (red). The color bar above the heatmap indicates different gene families or subfamilies. F, female; M, male; FB, fat body; H, head; M, midgut; O, ovary; SG, salivary gland; 1, 1 day; 11, 11 day

Digestive enzymes are directly related to the nutrients and energy uptake required for the development, metabolism, and reproduction of insects. Therefore, it is important to understand the genetics of digestive enzymes to develop new methods of biological control [25]. Genome-wide analysis shows that *A. suturalis* encodes genes for 20 alpha-amylases, 46 aminopeptidases, 45 carboxypeptidases, 65 cathepsins, 10 glucosidases, 62 lipases, 41 phospholipases, 31 polygalacturonase (PGs), and 197 serine proteases (SPs) (Fig. 6a and Additional file 2: Table S15). Compared with the other hemipteran insects, *A. suturalis* had a broad digestive enzyme spectrum, with a unique group of PGs and significantly expanded group of SPs and carboxypeptidases (Fig. 6a), which is consistent with *A. suturalis* that have a broad range of hosts and frequent host-switching behavior.

Among the various digestive enzymes in the salivary glands of mirid bugs, PG is one of the most important in the induction of visible plant injury. Notably, *A. suturalis* has a relatively small number of *PG* genes as compared with *A. lucorum*, but both species exhibit tandem genomic arrangement with high identity (71% *PGs* located on 4 clusters; Fig. 6b and Additional file 1: Figure S10). SPs are crucial proteolytic enzymes responsible for digestion and other processes including signal transduction and immune responses in insects. A total of 197 putative *SPs* were identified in the *A. suturalis* genome, which is slightly fewer than in *A. lucorum* (Fig. 6a). Phylogenetic tree analysis and sequence alignments of *SPs* in *A. lucorum* and *A. suturalis* suggest that the majority of *SP* genes evolved from tandem duplications and are arranged in clusters (Fig. 6c).

Interestingly, compared with *A. lucorum* and other insects, *A. suturalis* has significant expansion of carboxypeptidases genes, which have been found in various metazoan species and play important roles in diverse physiological and biochemical reactions [26]. Forty five members of the carboxypeptidase family were identified in the *A. suturalis* genome, these included 23 metal carboxypeptidases (seven metal carboxypeptidases containing the Peptidase_M2 domain and 16 metal carboxypeptidases containing the Peptidase_M14 domain) and 22 serine carboxypeptidases (12 serine carboxypeptidases containing the Peptidase_S10 domain and ten serine carboxypeptidases containing the Peptidase_S28 domain; Fig. 6d). Phylogenetic analysis showed that serine carboxypeptidase duplication and divergence led to the separation of Peptidase_S10 carboxypeptidases and Peptidase_S28 carboxypeptidases and that *A. suturalis* Peptidase_S10 carboxypeptidases have a very close genetic relationship with those of *A. lucorum* (Fig. 6d), though containing a smaller number of genes than *A. lucorum*.

The expression profile analysis of digestive enzyme genes in *A. suturalis* showed that *PGs* are specifically expressed and at high expression levels in salivary glands (Fig. 6e), with the same expression profile in *A. lucorum* [14]. This indicates that the salivary gland of *A. suturalis* has a very high ability to synthesize *PGs*. For *SP* genes, approximately 50% were specifically expressed in the fat body of males and the rest were expressed in diverse tissues, especially in the fat body and salivary gland (Fig. 6e). Meanwhile, the carboxypeptidases and other digestive enzyme genes in *A. suturalis* were mainly expressed in the midgut, fat body, and ovary, and likely function in digestion and degradation of toxic compounds from plants during the mesophyll feeding process, and may also provide essential nutrients for insect growth, development, and reproduction.

### Genetic basis of unique protein intake and long-distance migratory ability

**Fig. 7:**
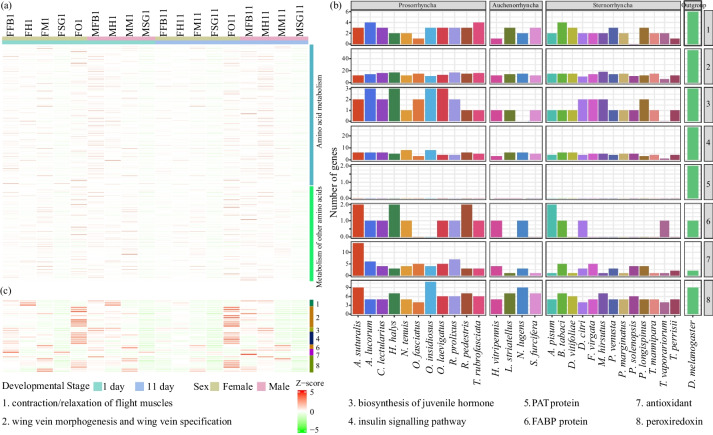
Evolution of genes putatively involved in energy consumption during *A. suturalis* flight. a Expression patterns of the amino acid metabolism and metabolism of other amino acid genes in different tissues and developmental stages of *A. suturalis*. b Stat of long-distance flight-associated genes in *A. suturalis*, other Hemiptera and *D. melanogaster*. **c** Expression levels of the putative candidate genes involved in long-distance flight of *A. suturalis*. female; M, male; FB, fat body; H, head; M, midgut; O, ovary; SG, salivary gland; 1, 1 day; 11, 11 day. Number one to eight in **b** and **c** represents the classification of genes involved in long-distance flight. PAT proteins, comprising of Perilipin, adipose differentiation-related protein (ADRP), tail-interacting protein, S3-12, and PLIN1-5. FABP, fatty-acid-binding proteins

One of the most distinguishing features of mirid bugs is their ability to prey on the aphid (*Aphis gossypii*) and other arthropod pests [5, 27]. Compared with feeding on plants alone, they have higher fecundity after feeding on aphids, and even fail to produce when feeding only on plants. The requirement by *A. suturalis* for feeding on aphids to complete a healthy life cycle is reflected in a higher intake demand for amino acids in this species [28]. Correspondingly, we identified 316 (1.6%) genes in *A. suturalis* and 288 (1.5%) genes in *A. lucorum* annotated to amino acid metabolism (09105) and 116 (0.6%) and 134 (0.7%) genes in the metabolism of other amino acids (09106) in *A. suturalis* and *A. lucorum* respectively. In contrast, only 209 (0.9%) and 94 (0.4%) genes for the two types of amino acid metabolism were found in the closest species *Nesidiocoris tenuis* (Fig. 3a). Transcriptome analysis showed that most of the above metabolism-related genes are specifically expressed in tissues other than the salivary gland (Fig. 7a), with higher expression in the fat body, supporting that the complex process of amino acid metabolism and high protein demand of mirid bugs.

The capacity for long-distance migration and broad range of host plants are the other distinguishing features of *A. suturalis*. Insect flight capacity depends on several factors, including wing and muscle morphology, neuroendocrine regulation and energy metabolism [29]. Here, we annotated genes that are potentially relevant to these morphological features and physiological processes as suggested by studies on the locust genome [29]. Through a comparison with other sequenced insect genomes belonging to the Hemiptera, we observed significant copy number expansion in genes associated with fatty-acid-binding proteins (*FABPs*), antioxidant and peroxiredoxin biosynthesis in the *A. suturalis* genome (Fig. 7b and Additional file 2: Table S16). FABPs contribute to the extremely high metabolic rate of energy generation [30] and antioxidant protection against reactive oxygen species damage caused by flight activity [31]. However, no *PAT* gene, which manages the access of lipases to lipid esters within the lipid droplet core, and which interacts with cellular machinery important for lipid homeostasis, was identified in any of the Hemiptera, in sharp contrast to the significant expansion in one of the world’s most destructive long-distance flight insects, the locust [29]. Transcriptome analysis shows that identified long-distance flight-associated genes are highly expressed in the fat body (Fig. 7c), which is the major site of energy metabolism in insects [32], indicating their important roles in the flying process. Considering a large amount of protein intake and the similarly high expression of amino acid metabolism-related genes in the fat body indicates that *A. suturalis* may be able to consume protein and fatty acid to generate energy at an extremely high metabolic rate to meet their intensive energy consumption during long-distance flight.

### Screening and identification of nineteen high-credibility candidate effectors in *A. suturalis*

**Fig. 8:**
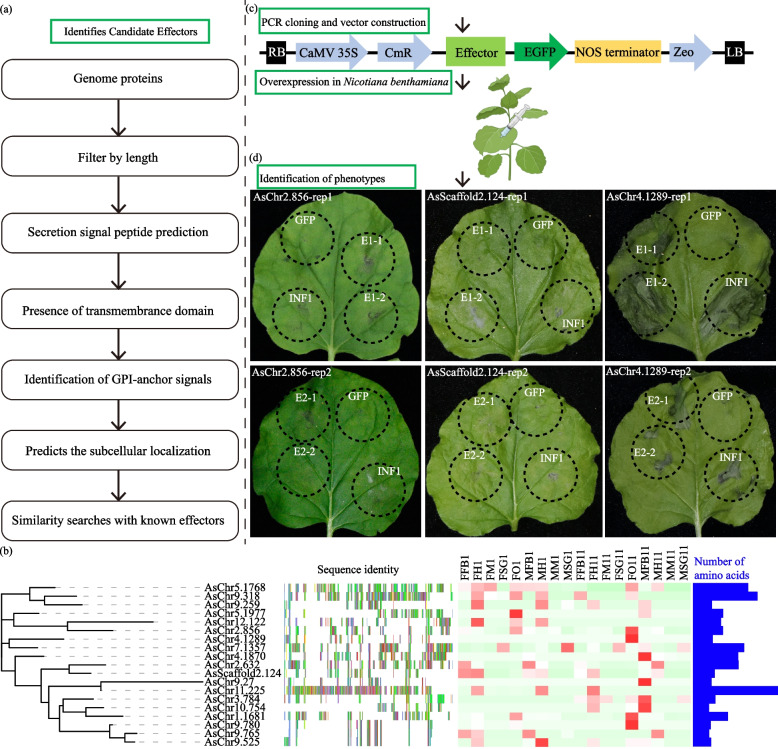
Identification of *A. suturalis* candidate effector proteins. **a** Bioinformatics pipeline for the identification of candidate effectors from *A. suturalis*. **b** Molecular characterization of candidate effectors. From left to right: phylogenetic tree, similarity alignment of amino acid sequence, expression levels in different tissues (each data block shows the *z*-score of FPKM value of the corresponding tissue organ) and number of amino acids. **c** Cloning and functional analyses of candidates to identify effector activities. Candidate effectors were overexpressed in *N. benthamiana* by agroinfiltration to determine whether they induce a cell death. **d** *Agrobacterium tumefaciens* infiltration assay for screening candidate effectors inducing cell death. Candidate effectors, the cell death-inducing gene *INF1*, and empty control (*GFP*) were expressed in *Nicotiana benthamiana* leaves via agroinfiltration. *GFP* is used as the negative control, and *INF1* is the positive control that induced cell death. There were two injection points on the same leaf, and two independent experiments were performed

Effectors are small molecules released by insects that interfere with the defense response of the host plant [33]. In order to explore potential candidate effectors and their functions in *A. suturalis*, we developed a functional genomics approach to identify candidate effectors from the high-quality assembly genome database of *A. suturalis* (Fig. 8a). A total of 19 candidate effectors genes were annotated based on features shared with plant pathogen/herbivore effectors with low identity of amino acid sequences and the number of amino acids ranging from 105 to 624 (Fig. 8b and Additional file 2: Table S17).

The most common feature of effector proteins is the induction of phenotypes in plants through cell death induction or suppression of pathogen-associated molecular pattern (PAMP)-triggered immunity (PTI) [34]. In insect, salivary proteins secreted by glands can induce reactive oxygen species (ROS) bursts and cell death at feeding sites by binding to specific enzymes in host plants [35]. Hence, to investigate the functions of predicted *A. suturalis* effectors in the plant, we performed transient overexpression of all 19 candidate effectors individually in *N. benthamiana* by agroinfiltration, screening for the induction of cell death phenotypes (Fig. 8c). The green fluorescent protein (GFP) gene was used as a negative control, and PAMP *INF1*, the most commonly used cell death inducer in plant immunity [36], was used as a positive control. After infiltration of candidate effectors into *N. benthamiana*, five candidate effectors out of the 19 and positive control *INF1*-induced cell death at 2 days post inoculation (dpi) were observed at the infiltration site. Among which, *AsChr2.856*, *AsChr4.1289*, and *AsScaffold2.124* induced the most obvious cell death phenotype in different batches of experiments. However, no cell death phenotype was observed for *GFP*, even at 7 dpi (Fig. 8d and Additional file 1: Figure S11). This provides evidence that the three candidate genes/proteins may be involved in the immune process during the plant–insect interaction. In the future, the identification and functional study of their downstream regulatory target genes in the main host plant, such as cotton, will provide important gene resources of cotton for the biological control against *A. suturalis*.

To investigate the functions of the candidate effector genes associated with feeding behaviors in *A. suturalis*, we carried out RNAi to silence *AsChr2.856*, *AsChr11.225*, *AsScaffold2.124*, and *AsChr4.1289* (Additional file 2: Table S18). The coding sequences of each gene were initially analyzed by blasting against the genome sequence of *A. suturalis*. A specific region that was unique to each gene was selected to design double-stranded RNA (dsRNA) for microinjection (Additional file 1: Figure S12a). In the RNAi analysis, the GFP was used as a negative control. After injecting the dsRNA into the nymphs of *A. suturalis*, qRT-PCR was performed to measure mRNA levels of each gene at 2 and 4 days (Additional file 2: Table S18). The results showed that mRNA accumulation for each gene was significantly reduced from days 2 and 4 post-injection compared to control GFP dsRNA-treated *A. suturalis* (Additional file 1: Figure S12b). Survival rates of *A. suturalis* fed on fresh green beans after dsRNA injection were recorded and showed significantly lower than that of dsGFP-treated insects starting at day 7 (Additional file 1: Figure S12c). Moreover, the body weight of dsAsChr2.856, dsAsChr11.225, dsAsScaffold2.124, and dsAsChr4.1289 injected *A. suturalis* was also significantly decreased from day 2, compared with those of GFP-injected controls (Additional file 1: Figure S12d). Additionally, honeydew secretion [37] was also used to determine the influence of AsChr2.856 and AsChr4.1289 on *A. suturalis* feeding, and found that the amount of honeydew excreted by *AsChr2.856* and *AsChr4.1289* silenced *A. suturalis* was significantly lower than that of GFP-silenced insects (Additional file 1: Figure S12e). In conclusion, our results demonstrated that the candidate effectors *AsChr2.856*, *AsChr11.225*, *AsScaffold2.124*, and *AsChr4.1289* facilitated the feeding performance of *A. suturalis*.

## Discussion

The rapid reproduction and wide geographical range of insects induce extremely high genomic heterozygosity, which has always brought great challenges to the assembly of their genomes. Here, by developing an inbred strain of *A. suturalis*, we were able to assemble the chromosome-scale reference genome through high-depth PacBio sequencing and highly complex assembly strategy, which combine Illumina short reads, PacBio long reads, 10x Chromium, and Hi-C technology. With the high-quality genome sequence of *A. suturalis*, we are able to comprehensively characterize the TEs and find an explosion of DNA and LINE transposons and the small local-scale gene duplications. TEs as a major component of metazoan genomes are associated with a variety of mechanisms that shape genome architecture and evolution [15]. In addition, the comparative genomic analysis of the *A. suturalis* and *A. lucorum* revealed extremely low genomic collinearity between them (Fig. 2a, b). This is likely due to their different genera and larger living habits [8], which may have led to genetic divergence over time. On the whole, these data complement available resources for genomic and evolutionary research of Miridae species.

In addition to effectors, a large number of digestive and detoxification enzymes, chemosensory receptors, amino acid metabolism components, and long-distance migratory genes are also important molecular players for *A. suturalis* to take nutrients and energy metabolism for adaptive survival. In the genome of *A. suturalis*, we observed the specific expansion and chromosome cluster arrangement of digestive enzyme (alpha-amylases, aminopeptidases, carboxypeptidases, cathepsins, glucosidases, lipases, phospholipases, *PGs*, and *SPs*) and detoxification protein genes (*CYPs*, *UGTs*, *GSTs*, *ABCs*, *CCEs*, and *PEBPs*), and the specific high expression in relevant tissues (Figs. 4 and 6). Notable are the detoxification subfamily of *CCEs* and ABCA subfamily of *ABCs*, and the *PG* gene encoding the plant polysaccharide digestive enzyme specific to *A. suturalis* and* A. lucorum* in the insects that have been sequenced in Hemiptera (Fig. 6) [14, 38]. Among these, phylogenetic tree analysis showed that eighteen *CCE* genes belong to the α-esterase clade, which is the only clade in the dietary/detoxification class that has been linked to lipid metabolism and xenobiotic detoxification [39], with two *A. suturalis*-specific branches (Fig. 4b), suggesting that *A. suturalis* may have enhanced the metabolism of endogenous compounds (hormones, pheromones, neurotransmitters) to detoxification of various xenobiotics than *A. lucorum*. Likewise, using siRNA injection-based RNAi, two *PGs* genes in *A. lucorum* have been demonstrated to elicit plant injury and knock-down significantly reduces the injury levels of cotton buds [40].

During insect feeding, plants perceive insect signals and trigger complex defense responses and the induction of hormonal pathways [41]. To enable successful feeding and infestation, insects also deliver a series of effectors from salivary glands into their host cells to suppress plant defense responses and modulate herbivore–plant interactions [33]. Several insect herbivore effectors hijacking plant signaling pathways have been described, for example the chaperonin GroEL from the *Myzus persicae* [42], vitellogenin protein from *Laodelphax striatellus* which effectively weakens H_2_O_2_-mediated plant defense [35], and Al6 of *A. lucorum* that functions as a glutathione peroxidase and suppresses ROS induced by pathogen-associated molecular pattern to inhibit pattern-triggered immunity (PTI)-induced cell death [36]. In this study, overexpression of novel identified candidate effectors of *A. suturalis* can induce cell death in *N. benthamiana* (Fig. 8), which preliminarily confirmed the biological functions of candidate effectors. Therefore, further work involving overexpression of these protein candidate effectors in the host plants will allow the identification of downstream regulatory genes and the molecular processes they perturb. This will provide important genetic resources for the biological control of *A. suturalis* and also provide new insight into the molecular mechanisms during insect-plant interactions.

## Conclusions

**Fig. 9:**
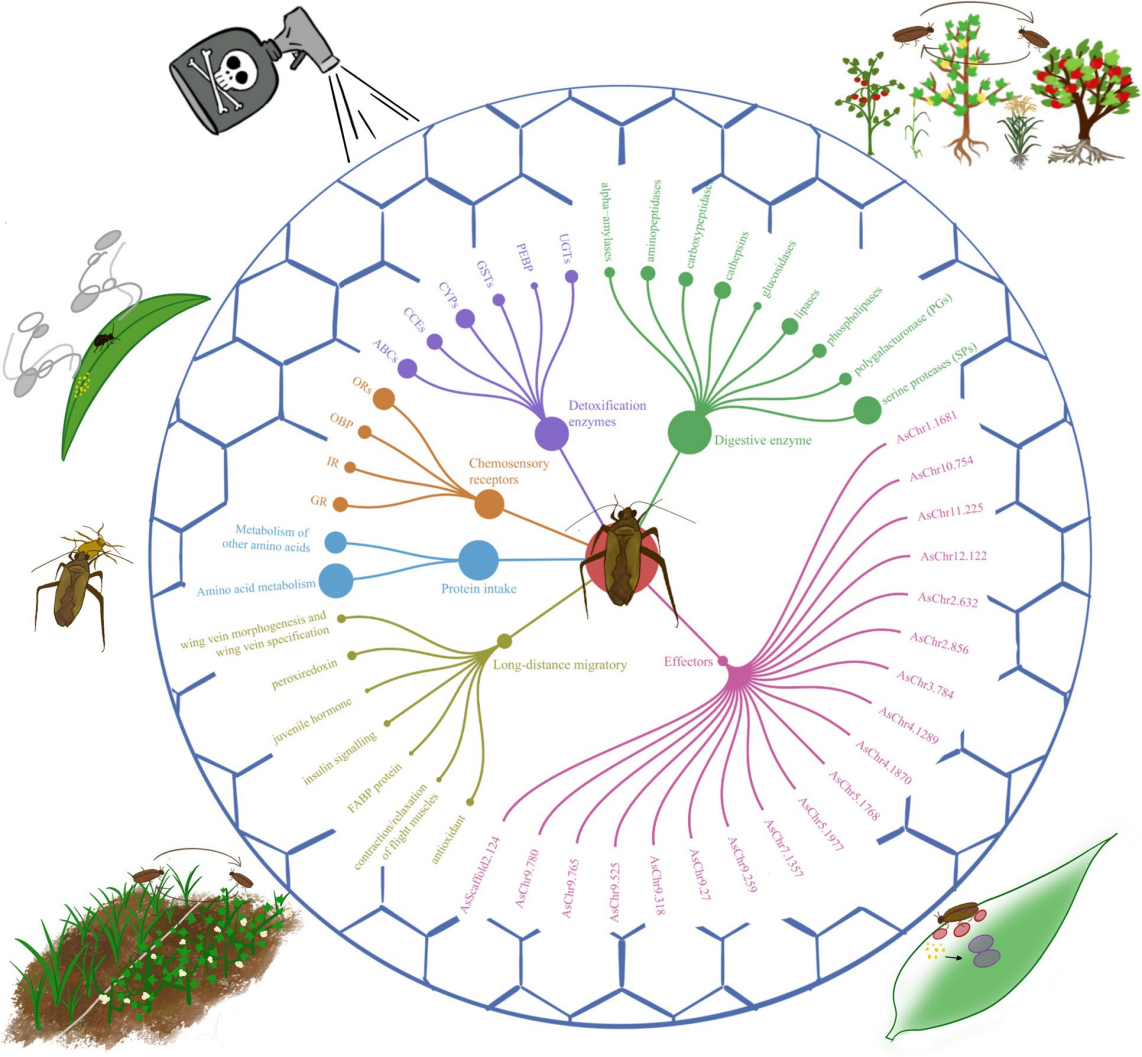
Graphic overview of candidate effectors, detoxification enzymes, and insecticide resistance, chemosensory receptors, digestive enzyme, protein metabolism, and long-distance migratory genes contribute to the omnivorousness, insecticide resistance, and survival adaptation mechanism of *A. suturalis*

In conclusion, the high-quality genomes of *A. suturalis* provide an opportunity for us to interpret its breadth range of omnivory, insecticide resistance, reproduction, and long-distance migratory characteristics (Fig. 9). Moreover, the diverse suites of detoxification genes and several classes of digestive proteinases provide *A. suturalis* with the metabolic plasticity needed to overcome the challenges of feeding on different host plants. Further studies on the functions and molecular mechanisms of these genes and candidate effectors will provide important genetic resources for the biological control of *A. suturalis*.

## Methods

### Insect rearing and genomic sequencing

*A. suturalis* nymphs and adults were collected from cotton fields at the Huazhong Agriculture University, Hubei Province, China. A laboratory colony was established and maintained at 26 ± 2°C, relative humidity (RH) 75 ± 5%, and 16:8 light: dark (L:D) and reared with fresh mung bean seedlings and cotton aphid (*Aphis gossypii*). An inbred strain of *A. suturalis* was developed by successive single-pair sib mating for 12 generations from this laboratory colony. This inbred strain was used for all genomic sequencing experiments in this study. For whole-genome sequencing and assembly, female nymphs with 5-day and adults from the above-inbred strain were collected and immediately frozen in liquid nitrogen.

### Library construction and sequencing

Genomic DNA from *A. suturalis* was extracted using a modified CTAB method. For Illumina sequencing, three paired-end libraries, with insert sizes of approximately 300, 500, and 700 bp, and three mate pair libraries, with insert sizes of 3, 5, and 8 kb, were constructed using standard Illumina protocols and sequenced on the Illumina HiSeq 2500 platform.

To enable an optimal assembly of the large and complex (high heterozygosity and repetitive DNA) reference genome, more than 70 Gb (> 260 × genome coverage and N50 of 30 kb) and 1.3 Tb (> 260 × genome coverage and N50 of 30 kb) of long sequence for *A. suturalis* was generated from SMRT cells on PacBio RS II and PacBio Sequel II platforms, respectively, following the manufacturer’s instructions.

To build superscaffolds, a library of 10x Chromium (10x Genomics, San Francisco, USA) was constructed. High molecular-weight (HMW) genomic DNA was used for library construction after passing quality assessment according to the manufacturer’s instructions without size selection. Sequencing-read libraries were sequenced using HiSeq 2500 with 2 × 150 paired-end reads to generate ~ 96 Gb (120 × coverage) raw data.

### Genome survey

To estimate the genome size, heterozygosity and repeat content, Jellyfish (v2.2.0) (https://github.com/gmarcais/Jellyfish) was used to generate a 21 K-mer frequency distribution. Depth of K-mer = 1 is considered as an error, and this error rate was used to calculate and correct the genome size. The formula for estimating the genome size is: Genome size = (K-mer num/main peak depth) × (1 − Error rate). The heterozygous ratio and repeat sequence ratio were estimated by the GCE (v1.0.2) [43].

### De novo assembly and polishing of contigs

A flowchart of contig, scaffold, and chromosome assembly in this study is shown in Fig. 1. Because of the high error rate of PacBio reads, we first corrected those by using an error correction module embedded in Canu (v2.0) with parameter correctedErrorRate 0.045 [44]. The high-quality PacBio sub-reads were then used for genome assembly by using NextDenovo (v2.3) (https://github.com/Nextomics/NextDenovo) to generate a draft genome assembly with default parameters. Following assembly, consensus polishing was run on all contigs using the Arrow software included with PacBio SMRTAnalysis (v5.1.0.26412; https://www.pacb.com/products-and-services/analytical-software/smrt-analysis/), using Minimap2 (v2.11) [45] and pbbamify for the read mapping stage. In addition, we also used NextPolish (v2.0) [46] to polish the draft genome with paired-end short reads to obtain the corrected genome. Due to the large assembly size observed, purge_dups (v2.3; https://github.com/dfguan/purge_dups) with parameters (-2 -a 50) was used to purge the haplotigs and error-containing fragments. This resulted in a purged primary assembly of total length 1.03 Gb, contig N50 of 785 kb, and a haplotig assembly of total length 936 Mb, contig N50 of 88 kb. These assemblies show no evidence of contamination by alignment with the UniVec and RefSeq microbial genome databases in the National Center for Biotechnology Information (NCBI).

To assess the completeness of genome assembly, we run BUSCO (v5.0.2) [47] using the insecta database (insecta_odb10), which contains 1658 conserved insecta genes, with parameters “-m genome –long.”

### Scaffolding by using mate pair and 10x Genomics library

The scaffolds was firstly built using Illumina mate-paired sequences via BESST (v2.2.4; https://github.com/ksahlin/BESST) [48] and then Racon (v1.4.21; https://github.com/lbcb-sci/racon) was used to generate a consensus assembly using the corrected PacBio long-read data.

The Linked-reads from the 10x Genomics library were mapped to the consensus assembled scaffolds using BWA-MEM with a default parameter, and clusters of reads with the same barcode mapped to adjacent contigs in the scaffolds were identified as being part of a single long molecule. Then, ARCS (v1.2.2; https://github.com/bcgsc/arcs) and LINKS (v1.8.7; https://github.com/bcgsc/LINKS) pipeline was used to extend consensus scaffolds into supscaffolds and composed into the final scaffold output of assembly.

### Chromosome assembly using Hi-C

For the Hi-C sequencing and scaffolding, the fresh tissues from living insects were fixed with paraformaldehyde and lysed, and the cross-linked DNA was then digested with DpnII (NEB) restriction endonuclease. Hi-C DNA recovery and subsequent DNA manipulations were performed following [49]. Then a Hi-C library was sequenced on an Illumina NextSeq instrument with 2 × 150 bp reads.

For chromosome-level assembly, these clean data from Hi-C library were trimmed to remove low-quality bases and Illumina adapter sequences using trim-galore (v0.6.5; https://www.bioinformatics.babraham.ac.uk/projects/trim_galore/), and then checked with HiCUP [50]. The clean Hi-C read pairs were aligned to consensus supscaffolds described above using juicer (v0.6.5) and 3D-DNA (v0.6.5) with a default parameter to anchored and oriented the supscaffolds to pseudochromosomes on the basis of the Hi-C contact map. Finally, for each chromosome cluster, we inspect and manually correct with Juicebox assembly tools (v2.7.8; https://github.com/aidenlab/Juicebox).

To further improve the accuracy of reference assembled contigs, two-step polishing strategies were performed: we first used PacBio long reads and carried out gap filling with PBJelly [51] and then used highly accurate Illumina paired-end reads to further correct the base errors with Pilon (v1.20) [52].

To evaluate the quality of the genome assembly, the Illumina sequencing reads were mapped using Bowtie2 (v2.3.5) [53]. To evaluate the completeness of genome assemblies, the 1614 conserved protein models in the insecta_odb10 dataset were searched against both genomes by using the BUSCO (v5.0.2) program [47] with the –long parameter.

### RNA-seq library construction, sequencing, and analysis

Total RNA was extracted from head, midgut, salivary gland, and fat body samples from female and male of *A. suturalis*. For female, the ovary tissue was also used to extract total RNA. After DNase treatment, RNA-seq libraries were constructed and sequenced on the Illumina HiSeq 2500 platform with 150 bp paired-end sequences according to the manufacturer’s recommended protocol.

The RNA sequencing reads were trimmed, aligned, and quantified using Trim Galore (v0.4.5; https://github.com/FelixKrueger/TrimGalore/), Hisat2 (v2.2.1) [54] and Stringtie (v2.1.4) [55], respectively, with default parameters. We measured the gene expression level by FPKM and TPM (transcripts per million).

### Genome annotation: repetitive sequences, gene models and noncoding RNA

The annotation of repetitive DNA followed both homology-based prediction and de novo identification of repeats as previously described [49]. In brief, TRF (v4.07b) [56] and MISA [57] were used to identify tandem repeats and simple sequence repeats (SSRs). Long terminal repeats (LTRs) were identified using LTR_retriever [58] on the basis of the results of LTRharvest [59] and LTR_Finder [60] with the suggested parameters described in the manual. RepeatMasker (v4.0.5) [61] were utilized to mask the genome using known transposons (a de novo repeat library of *A. suturalis* that built by RepeatModeler), and Extensive de novo TE Annotator (EDTA) [62] was used for comprehensive TE identification. All aforementioned results were combined and merged to generate a non-redundant list of repeat elements that reside in the genome.

Gene models were annotated based on ab initio gene predictions, homology support, and RNA sequencing evidence using genome that all repetitive regions have been soft-masked. For ab initio prediction, Augustus (v3.4.0) [63], GlimmerHMM (v3.04) [64], GeneMark-ES (v4.69) [65], and SNAP (v2006-07–28) [66] were used for de novo-based gene prediction with the default parameters. The homology EST and protein databases were constructed by integrating the Hemiptera species from the NCBI databases and SwissProt databases. Additionally, filtered proteins (incomplete and wrong) of *A. lucorum* [14] and *Acyrthosiphon pisum* were used for homology-based prediction with GeMoMa (v1.7.1) [67] using the default settings. Then, RNA sequencing transcripts assembled with HISAT and StringTie were used to assist in gene structure predictions. Finally, all predictions were integrated to produce a consensus gene set using EVidenceModeler (v1.1.1) [68]. To assess the completeness of the gene set, BUSCO (v5.0.2) [47] was used to evaluate the gene set based on the encoded proteins using insecta_odb10.

Gene functional annotations were assigned by aligning protein sequences to SwissProt and TrEMBL databases using BLASTP (*E*-value (expected value) ≤ 1 × 10^−5^). Motifs and domains were searched using InterProScan (v5) [69] against all default protein databases including ProDom, PRINTS, PfamA, SMART, TIGRFAM, PrositeProfiles, HAMAP, PrositePatterns, SITE, SignalP, TMHMM, Panther, Gene3d, Phobius, Coils, and CDD. The gene pathways of the predicted sequences were extracted from the Kyoto Encyclopedia of Genes and Genomes (KEGG) Automatic Annotation Server (KAAS, v2.1).

Noncoding RNA species including miRNA, tRNA, rRNA, and snRNA were annotated using several methods. tRNA species were predicted using tRNAscan-SE (v1.3.1) [70] with default parameters. miRNA and snRNA were identified by searching against the Rfam database (release 12.0) using Infernal (v1.1.1) [71] with default parameters.

### Gene family and phylogenetic analysis

In order to achieve a robust phylogenetic reconstruction with high confidence and concordance, orthologous groups were constructed using the proteome sequences of *A. suturalis* and 27 sequenced hemipteran insects, as well as *Drosophlia melanogaster* as an outgroup. All mentioned genomic resources were downloaded from the InsectBase 2.0 database (http://v2.insect-genome.com/Genome). ORFs with premature stop codons or encoded less than 50 amino acids were removed. Then, the longest proteins for each species were collected and aligned with each other by all-versus-all BLASTP (v2.2.28) with an *E*-value of 1E^−5^. OrthoMCL [72] was employed to identify 34,635 orthologs and paralogs for all species with the parameter (− *I* = 1.5). To construct the phylogenetic tree of *A. suturalis* and other 27 hemipteran insects, we collected 65 single-copy gene families with orthologs and aligned the orthologs of each family using MAFFT (v7.471) [73], then low-quality regions were trimmed using Gblocks (v0.91b) [74]. A maximum likelihood phylogenetic tree was constructed using concatenated alignment with RAxML (v8.2.1264) and the PROTGAMMAILGF model to automatically determine the best reasonable tree by conducting 1000 bootstrap replicates. The concatenated alignment and maximum likelihood tree that used as a starting tree were input into MCMCtree program [75] to estimate species divergence time. Calibration times were set according to a previous paper, minimum = 320 Ma and maximum = 390 Ma for *D. melanogaster* and *A. lucorum* [76]. The Markov chain Monte-Carlo analysis was repeated 10,000,000 times with 1000 steps.

We identified the expansion and contraction of orthologous groups using computational analysis of gene family evolution CAFE (v4.2) [77] according to the difference in gene number of each orthologous group of each species with parameters: -p 0.05 -t 1 -r 10,000. To determine significance for expansion and contraction of orthologous groups, a family-wise *p*-value (based on a Monte-Carlo re-sampling procedure) was calculated and the threshold for significant expansion and contraction was set to *P*-value < 0.05.

### Genome alignment and gene synteny analysis

Genome alignment either inter-species or intra-species of *A. suturalis* and *A. lucorum* was performed using the minimap2 (v2.16-r922) [45] program with parameter settings -x asm5 and dot plot was plotted using dotPlotly (https://github.com/tpoorten/dotPlotly) to display the synteny block located in inter-chromosomal or intra-chromosomal.

Syntenic gene pairs between *A. suturalis* and *A. lucorum* were identified with JCVI (v0.84) [78]. Using coding sequence (CDS) and annotation gff3 files as input data, the syntenic blocks for each pair species were identified through “jcvi.compara.catalog ortholog” with a parameter of–cscore = 0.8. The syntenic blocks were filtered using “jcvi.compara.synteny screen” with parameters:–minspan = 30–simple. Synteny pattern was detected by “jcvi.compara.synteny depth–histogram.”

### Functional genomics approach identifies candidate effectors from *A. suturalis*

The bioinformatics for the identification of *A. suturalis* candidate effectors was modified based on a pipeline for the prediction of aphid effectors [79]. In brief, protein sequences with less 1000 amino acids were selected for future analysis. We then applied the SignalP (v5.0) and TMHMM (v2.0) to predict the presence of signal peptides and transmembrane domains with a default parameter. Protein sequences that in addition to the signal peptide also contained no transmembrane domain were scanned for GPI-anchored proteins using GPI-SOM [80]. Proteins that were highly probably and probable of having a GPI anchor were discarded. Thus, using bioinformatics, proteins with signal peptides, with two or less transmembrane domains and without GPI anchors were considered for possible secretion. To identify candidate effector, the insect effector sequences were retrieved from publications [36, 79, 81] and used as queries for Blast searches (*E*-value < 1 × 10^–5^) against above *A. suturalis* secreted proteins. Additionally, prediction of *A. suturalis* candidate effector subcellular localization was performed using the Deeploc (v1.0) [82].

### Analysis of the digestive enzyme, chemosensory receptor, detoxification enzyme, and long-distance migratory genes

Detoxification and insecticide resistance-related genes, including cytochrome P450s (PF00067), glutathione S-transferases (GSTs; PF02798 and PF00043), carboxylesterases (PF00135), uridine diphosphate (UDP)-glucosyltransferases (PF00201), ABC transporters (PF00005), and phosphatidylethanolamine-binding protein (PEBP; PF01161) gene family, were identified in *A. suturalis* candidate and other hemipteran insect genomes based on Pfam domains contained in their protein sequences using hmmsearch in HMMER (v3.0) [83]. The classification and annotation of corresponding gene families were performed based on sequence similarities to *D. melanogaster* sequences from the FlyBase database (http://flybase.org/) [84].

For the genes of chemosensory receptor (PF08395), including encoding odorant-binding protein (OBP), odorant receptors (ORs), gustatory receptor (GR) and ionotropic receptor (IR) gene family, and long-distance migratory were identified in *A. suturalis* candidate and other hemipteran insect genomes based on searched candidate sequences in the genome assembly using BLASTP [85] (sequence similarity ≥ 80% and *e*-value ≤ 1e − 5) with a set of reference sequences obtained from InsectBase (v2.0) [86], FlyBase (http://flybase.org/) and NCBI.

The Simple Modular Architecture Research Tool (SMART) and conserved domain databases were used to examine all candidate genes and genes with incomplete domains were removed.

For evolutionary analysis of genes family, we aligned protein sequences using MAFFT (v7.471) [73] with E-INS-I iterative refinement method and automatically trimmed by trimAl (v1.1) [87]. The alignment was then used to construct maximum likelihood phylogenetic tree using FastTree (v2.1) [88].

### Agrobacterium tumefaciens infiltration assay for screening candidate effectors

The candidate effector cDNAs were amplified from isolated total RNA of *A. suturalis*, and the signal peptides were removed for insertion into the EGFP-pGWB405 vector with the control of the 35S promoter. The successful constructed plasmids were then transformed into *A. tumefaciens* strain GV3101 by electroporation. Recombinant *A. tumefaciens* strains were cultured (28℃ in Luria–Bertani media using Zeocin antibiotics), washed, and re-suspended in infiltration buffer (10 mM MgCl_2_, 500 mM MES, 100 mM acetosyringone, pH = 5.6) until an appropriate optical density (OD) of 0.4 at 600 nm was reached to harvest for infiltration. Agroinfiltration assays were performed using a needleless syringe on 4–6-week-old *N. benthamiana* plants. For the induction of cell death assays, a well-known PAMP from the plant pathogen Phytophthora infestans, *INF1*, was used as a positive control, while empty vector with GFP was the negative control. Each strain was assayed on two replicated plants. Symptom development of the injected leaves was photo-recorded regularly. All the primers used for PCR amplification were synthesized by GenScript (Nanjing, China).

### RNA interference and feeding-bioassay for *A. suturalis*

In this experiment, double-stranded RNA was synthesized using a previously described method [89]. The GFP dsRNA was also synthesized using the same method as the negative control. Then, microinjector was used to inject 50 nl dsRNA (15,000 ng/μl) into the third instar nymphs. After 2 and 4 days of injection, total RNA was extracted from the nymphs and qRT-PCR were performed using previous methods [89] to detect the silencing efficiency. Relative gene expression levels were determined using QEF1γ levels as internal controls. The means and standard deviations were calculated from data obtained from three replicates. All the above primers used for PCR amplification are listed in Additional file 2: Table S18 and synthesized by GenScript (Nanjing, China).

To observe the survival status of *A. suturalis*, fresh green beans were placed in a cage with 10 injected *A. suturalis*. The survival rate was recorded daily for a week, which was calculated by dividing the number of surviving insects per group by the total number of treatments per group. To measure the weight change of *A. suturalis* after injection, they were put into a cage containing fresh green beans 2 days after injection. The body weight of the larvae was recorded at the beginning (day 0) of the assay and on day 2 for all groups. The weight gain rate was calculated by dividing the number of pounds gained by the initial weight. In addition, the honeydew content on the filter paper was measured using the previously described method [90]. Five *A. suturalis* nymphs injected for 2 days were placed in a plastic petri dish with filter paper and fresh green beans. The filter paper was collected and soaked in 0.1% (wt/vol) ninhydrin solution after 2 days of feeding. After 30 min of drying in an oven at 60°C, the filter paper turned purple or showed purple spots due to the content of amino acids in honeydew.

### Supplementary Information

**Additional file 1: Note 1. Figure S1. **Harm of *A. suturalis* to cotton field.** Figure S2. **19-mers depth distribution of 308 Gb raw sequence data from libraries with insert size 300 bp. **Figure S3. **BUSCO Assessment of *A. suturalis* genome using insecta _odb10. **Figure S4. **Hi-C map of the *A. suturalis* genome showing genome-wide all-by-all interactions. **Figure S5. **Distribution of divergence rate of different types of TEs in the *A. suturalis* genome.** Figure S6.** Permutation test examines the correlation between the density of genes and TEs. **Figure S7. **Distribution of 86 *CYP* genes in the *A. suturalis* chromosomes. **Figure S8. **Distribution of 35 *UGT* genes in the *A. suturalis* chromosomes. **Figure S9. **Distribution of 55 *CCE* genes in the *A. suturalis* chromosomes. **Figure S10. **Distribution of 31 *PG* genes in the *A. suturalis* chromosomes. **Figure S11. **Identification of *A. suturalis* candidate effector proteins. **Figure S12. **RNAi of *Chr2.856*, *Chr11.225*, *Scaffold2.124* and *Chr4.1289* effects on feeding behaviour for *A. suturalis*.

**Additional file 2: Table S1.** Summary of Illumina reads (DNA) for *A. suturalis*. **Table S2.** Summary of PacBio reads for *A. suturalis*. **Table S3.** Summary of 10x Chromium Linked-reads for *A. suturalis*. **Table S4.** Summary of Hi-C reads for *A. suturalis*. **Table S5.** Summary of *A. suturalis *genome assembly. **Table S6.** Summary of Illumina reads mapping to *A. suturalis* genome. **Table S7.** Assessment of the genome assembly completeness by BUSCO. **Table S8.** Assessment of the genome annotation completeness by BUSCO. **Table S9.** Relative amounts of the major TE families in *A. suturalis* genome. **Table S10.** GO enrichment analysis of orthologous genes in *A. suturalis* and *A. lucorum*. **Table S11.** KEGG enrichment analysis of orthologous genes in *A. suturalis* and *A. lucorum*. **Table S12.** KEGG enrichment analysis of gene families that are unique to *A. suturalis*. **Table S13.** Gene number comparison of detoxification enzymes and insecticide resistance genes in *A. suturalis*. **Table S14.** Gene number comparison of chemosensory receptor genes in *A. suturalis*. **Table S15.** Gene number comparison of digestive enzyme genes in *A. suturalis*. **S16.** Gene number involved in energy consumption during *A. suturalis *flight. **Table S17.** Functional annotation of candidate effectors for *A. suturalis*. **Table S18.** Primers used for dsRNA amplification and relative expression detection.

## Data Availability

The *Adelphocoris suturalis* assembly and annotation data are available at Assembly database (GCA_030762985.1) of NCBI [91], figshare (https://figshare.com/s/51e208e3a147107084ef) [92] and Jinlab (http://jinlab.hzau.edu.cn/download/ and http://jinlab.hzau.edu.cn/InsectDB/Adelphocoris_suturalis/metadata/). The raw sequencing data used for de novo whole-genome assembly are available from the BioProject under accession number PRJNA874802 [93]. Transcriptome data of Illumina RNA-seq is available at the BioProject under accession number PRJNA869339 [94]. Further details on data accessibility are outlined in the supplementary materials and methods.

## Abbreviations

RNAi: RNA interference
FARs: Fatty acyl-CoA reductases
Hi-C: High-throughput chromosome conformation capture
BUSCO: Benchmarking Universal Single-Copy Orthologs
TEs: Transposable elements
LTR: Long terminal repeat
GO: Gene ontology
KEGG: Kyoto Encyclopedia of Genes and Genomes
WGD: Whole-genome duplication
CYPs: Cytochrome P450s
UGTs: UDP-glucuronosyltransferases
GSTs: Glutathione S-transferases
ABCs: ATP-binding cassette transporters
CCEs: Carboxylesterases
PEBP: Phosphatidylethanolamine-binding protein
OBP: Odorant-binding protein
OR: Odorant receptor
GR: Gustatory receptor
IR: Ionotropic receptor
PGs: Polygalacturonase
SPs: Serine proteases
FABPs: Fatty-acid-binding proteins
PTI: (PAMP)-triggered immunity
ROS: Reactive oxygen species
GFP: Green fluorescent protein
dsRNA: Double-stranded RNA
