# Supplementary material for: The chromosome-scale reference genome of mirid bugs (Adelphocoris suturalis) genome provides insights into omnivory, insecticide resistance, and survival adaptation
Source: BMC Biol. 2023 Sep 19;21:195. doi: 10.1186/s12915-023-01666-3 (PMC10510153; doi:10.1186/s12915-023-01666-3)
Supplement: Supplementary file 1 — Additional file 1: Note 1. Figure S1. Harm of A. suturalis to cotton field. Figure S2. 19-mers depth distribution of 308 Gb raw sequence data from libraries with insert size 300 bp. Figure S3. BUSCO Assessment of A. suturalis genome using insecta _odb10. Figure S4. Hi-C map of the A. suturalis genome showing genome-wide all-by-all interactions. Figure S5. Distribution of divergence rate of different types of TEs in the A. suturalis genome. Figure S6. Permutation test examines the correlation between the density of genes and TEs. Figure S7. Distribution of 86 CYP genes in the A. suturalis chromosomes. Figure S8. Distribution of 35 UGT genes in the A. suturalis chromosomes. Figure S9. Distribution of 55 CCE genes in the A. suturalis chromosomes. Figure S10. Distribution of 31 PG genes in the A. suturalis chromosomes. Figure S11. Identification of A. suturalis candidate effector proteins. Figure S12. RNAi of Chr2.856, Chr11.225, Scaffold2.124 and Chr4.1289 effects on feeding behaviour for A. suturalis. [file 12915_2023_1666_MOESM1_ESM.docx]

## Table of Contents

## Note 1. Expansion and widespread expression of detoxification enzymes gene family in *A. suturalis*

The enhancement of detoxification metabolism is the major mechanism of insect resistance to insecticides. The sequencing of the genome of *A. suturalis,* a highly polyphagous agricultural pests, gives us the opportunity to master the detoxification enzymes related to resistance and study the biochemical mechanism of metabolic resistance of *A. suturalis* to insecticides and plant toxic secondary metabolite such as terpene, alkaloids and flavonoids. Several enzyme families implicated in detoxification were identified in the *A. suturalis* genome, including cytochrome P450s (*CYPs*), UDP-glucuronosyltransferases (*UGTs*), glutathione S-transferases (*GSTs*), ATP-binding cassette transporters (*ABCs*), carboxylesterases (*CCEs*) and phosphatidylethanolamine-binding protein (*PEBP*) (Figure 4).

### 1.1 Cytochrome P450s (*CYPs*)

A total of 86 P450s containing five *CYP2*, 51 *CYP3*, 27 *CYP4*, and three *CYPMito* have been identified in the genome of *A. suturalis*, among which CYP3 shows an expansion relative to most close species *A. lucorum*. Members of the CYP3 clade have been implicated in the oxidative detoxification of plant secondary metabolites and synthetic insecticides [1, 2]. The phylogenetic tree of CYPs exhibited three *A. suturalis*-specific branches in the CYP3 clade (Figure 4b), suggesting that CYP3 undergo experienced species-specific expansion to enhance the detoxification activity in *A. suturalis*. What’s more, most *CYPs* genes show tandem duplications, with several chromosomes in the *A. suturalis* genome assembly possessing two or more CYP loci that are closely related to one another, and the largest CYP cluster was located on Chr3 and belong to CYP3 clade (Additional file 1: Figure S7). The species-specific expansion and tandem duplications of *CYPs* may offer *A. suturalis* stronger detoxification ability and insecticide resistance. Meanwhile, the RNA-Seq data covered major developmental stages and tissues of the *A. suturalis*, were applied to investigate the expression profile of all identified *CYP* genes. All 86 *CYP* genes were detected and classed into expressed genes. Moreover, the *CYP3* and *CYP4* gene families tended to be expressed in the midgut of both sexually immature (1 day old) and sexually mature (11 days old) females and males (Figure 4c). Meanwhile, we found that all these *CYP* genes tended to be widely expressed at major developmental stages and in all tissues, except for relatively low expression in the salivary gland, suggesting the importance of *CYP* genes in all life stages for *A. suturalis* to detoxify the plant xenobiotics.

### 1.2 UDP-glucuronosyltransferases (*UGTs*)

UGTs are a kind of multifunctional superfamily enzymes widely existing in animals, plants, bacteria and viruses. Their glycosylation plays a very important role in inhibiting and metabolizing the toxicity of a variety of endogenous and exogenous compounds. Insects can use the glycosylation activity of UGT enzyme to degrade the toxic substances invading the body. Therefore generate resistance to pesticides. Total 35 putative *UGTs* were manually annotated in the *A. suturalis* genome, which is greater than in most other species of Prosorrhyncha (Hemiptera suborder, only 19 in *A. lucorum*) (Figure 4a). Moreover, approximately 91 % of *A. suturalis* *UGTs* are arranged in a tandem manner and 13 of them were concentrated in one cluster of Chr5 (Additional file 1: Figure S8). Phylogenetic tree showed that the largest *UGT* family observed in *A. suturalis*. UGT397, is consists of 22 genes and exhibits a significant *A. suturalis*-specific expansion compared with *A. lucorum* (Figure 4b). The expansion of UGTs indicates that it may exhibit strong detoxification and offer insecticide resistance in *A. suturalis*. Consistent with this hypothesis, all *UGT* genes were widely expressed across different tissues and developmental stages of the *A. suturalis*, especially strongly express in the midgut (Figure 4c), which may help *A. suturalis* adapt to feeding on different host species that produce different defensive compounds.

### 1.3 Glutathione S-transferases (*GSTs*)

GSTs mainly catalyzes the covalent conjugation of glutathione with toxic electrophilic and hydrophobic substrates, so as to participate in a variety of detoxification metabolic processes in insects [3]. Additionally, 30 *GSTs* were identified in *A. suturalis* (Figure 4a). Phylogenetic analysis of the *GSTs* in *A. lucorum* and *A. suturalis* showed similar evolutionary branches (Figure 4b). Expression profile analysis showed that most *GSTs* genes were expressed in various tissues, especially high expression in the midgut is in line with their detoxification function (Figure 4c). Both phylogenetic and expression analysis suggests the *GSTs* experienced a recent species-specific expansion in *A. lucorum* and *A. suturalis*, enabling better detoxification of toxic substances and adaptation to the environment.

### 1.4 ATP-binding cassette transporters (*ABCs*)

ABCs form the largest family of transmembrane proteins, which bind and hydrolyze ATP and use the energy of this reaction to drive the transport of various substrates across cellular membranes, and play the role of detoxification, defense and protection to various tissues and organs [4]. A total of 90 *ABCs* genes were found in the genome of *A. suturalis* and is more than other species of Hemiptera (Figure 4a). According to the homology of nucleotide-binding domains (NBDs), which is the components of their ATP-binding domain(s), 44 *ABCs* were grouped into the 8 families (33, 9, 13, 3, 4, 8, 11 and 9 ABCs proteins belonging to the ABCA-H subfamilies), among which ABCA was the largest subfamilies (Figure 4b). The ABCA subfamily may be involved in lipid metabolism and promote the development of wings and elytra or provide energy for flight [5, 6]. Therefore, the large expansion of ABCA subfamily in *A. suturalis* (Figure 4b) is consistent with its strong migration ability. The spatial expression profiles of these ABC transporter genes were estimated by analyzing the FPKM values and shows that 51% genes were widely expressed in sampled tissues of *A. suturalis*. The expansion and expression of ABCs superfamily in *A. suturalis* play a very important role in pesticide resistance and overcoming multiple chemical plant defenses by inhibiting the accumulation of intracellular pesticides and their metabolites [7].

### 1.5 Carboxylesterases (*CCEs*)

CCEs is one of the important substances for insects to resist exogenous pesticides. It plays an important role in the resistance of commonly used organophosphorus and carbamate, and can make insects produce metabolic resistance. A total of 55 *CCEs* genes were detected from the genome data of *A. suturalis* (Figure 4a). The constructed phylogeny tree showed that all the *CCEs* fall into three main phylogenetic classes of dietary/detoxification, hormone/semiochemical processing and neuro/developmental functions (Figure 4b). Among these, eighteen *CCEs* genes belong to the α-esterase clade, which is the only clade in the dietary/detoxification class that has been linked to lipid metabolism and xenobiotic detoxification[8], and are major arranged in a tandem manner in Chr5 (Additional file 1: Figure S9). Moreover, the phylogenetic tree of *CCEs* also exhibited two *A. suturalis*-specific branches (Figure 4b), suggesting that *A. suturalis* may have enhancing the metabolism of endogenous compounds (hormones, pheromones, neurotransmitters) to detoxification of various xenobiotics than *A. lucorum*. Eighteen *CCEs* genes were involved in the hormone/semiochemical processes, which were catalytic enzymes with crucial roles in hormone/pheromone olfactory processes, reproductive behaviors and xenobiotics metabolism [9], with 8 juvenile hormone esterase, 3 β-esterases and 7 integument esterases. The remaining 19 *CCEs* genes were located in the neuro/developmental processes class, with 2 acetylcholinesterase, 3 gliotactins, 12 neuroligins and 2 glutactins. Within this subfamily, acetylcholinesterase is known to be involved in OP resistance in insects [10], other clades were non-catalytically adhesive proteins implicated in cell-to-cell interactions and signal transductions in the nervous system [11]. The tissue-specific expression pattern of *CCEs* genes in *A. suturalis* show that all *CCEs* genes from neuro/developmental processes class have significantly upregulated expression in the head than in other tissues. However, other *CCEs* genes from dietary/detoxification and hormone/semiochemical were highly expressed in the midgut and fat bodies (Figure 4c). The considerable number of CCEs and abundantly distributed in fat bodies and the midgut indicates their potentially important roles in detoxifying insecticides and thus conferring insecticide resistance in *A. suturalis*.

### 1.6 Phosphatidylethanolamine-binding protein (*PEBP*)

PEBPs are a highly conserved group of proteins with associated various biological processes and have been reported to contribute to insect resistance to pesticides in *B. tabaci* [12]. However, only seven and five *PEBPs* were contained in the genome of *A. suturalis* and its related species *A. lucorum* (Figure 4a), which is far lower than 202 in the *B. tabaci* genome[12]. Phylogenetic tree of *PEBPs* in *A. suturalis*, *A. lucorum* and *B. tabaci* shows that *PEBPs* in *A. suturalis* and *A. lucorum* is on different evolutionary branches of *B. tabaci*, but have the same evolutionary process with each other (Figure 4b). Moreover, transcriptome analysis showed that PEBPs in *A. suturalis* were major highly expressed in the head and fat bodies than in other tissues (Figure 4c). PEBPs are a highly conserved group of proteins and associated with various biological processes. Compared with *B. tabaci*, the small number of *PEBPs* genes in *A. suturalis* may indicate that *A. suturalis* uses a different defense strategy from *B. tabaci* for pesticide metabolism and detoxification mechanisms.

In summary, the expansion of detoxification gene families and their wide expression in different tissues at different developmental stages in *A. suturalis* provides a solid genetic basis for its well-known insecticide resistance and its ability to occupy a broad range of host plants with a diversity of defenses.

## 2. Supplementary Figure


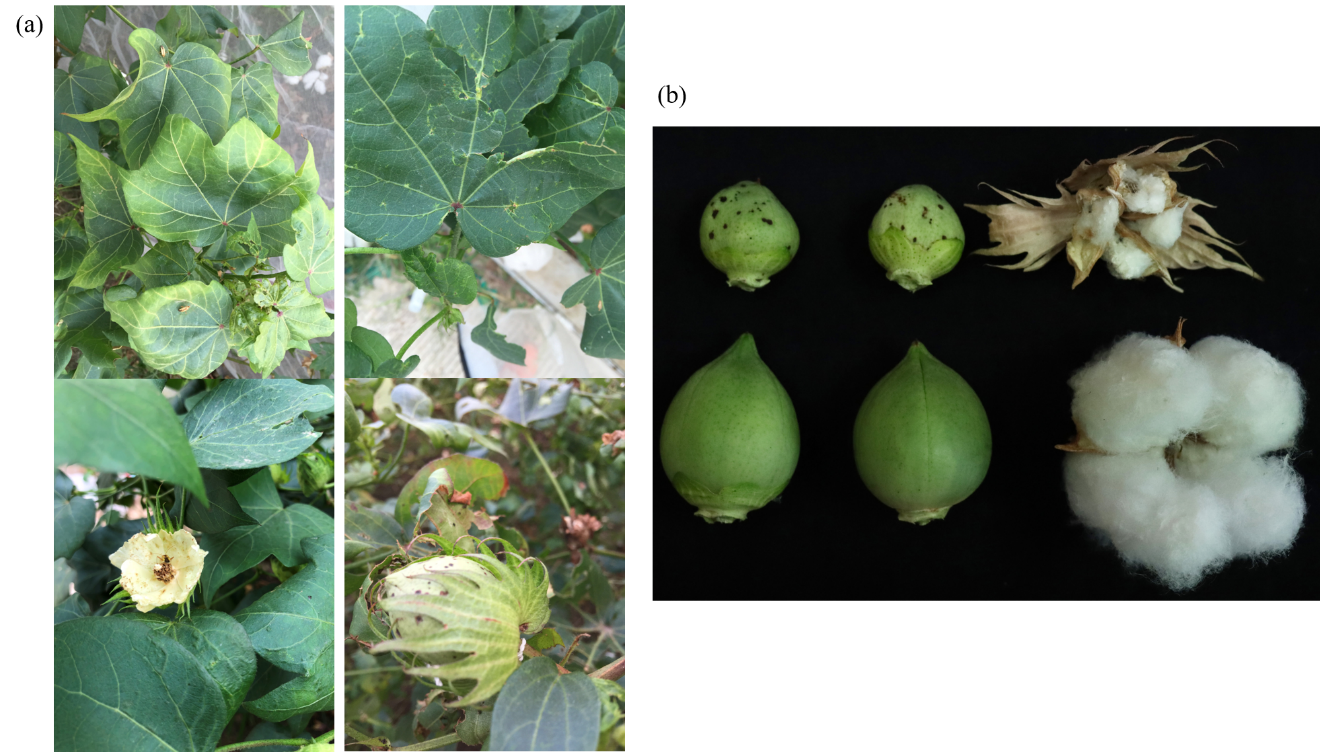


**Supplementary Figure S1. Harm of *A. suturalis* to cotton field. a,** Damage of black stink bug to cotton leaves, flowers and peaches in the field. **b,** After feeding of cotton boll by the *A. suturalis*, reduction in boll size, formation of black spots, developmental abnormality and cracking.

**
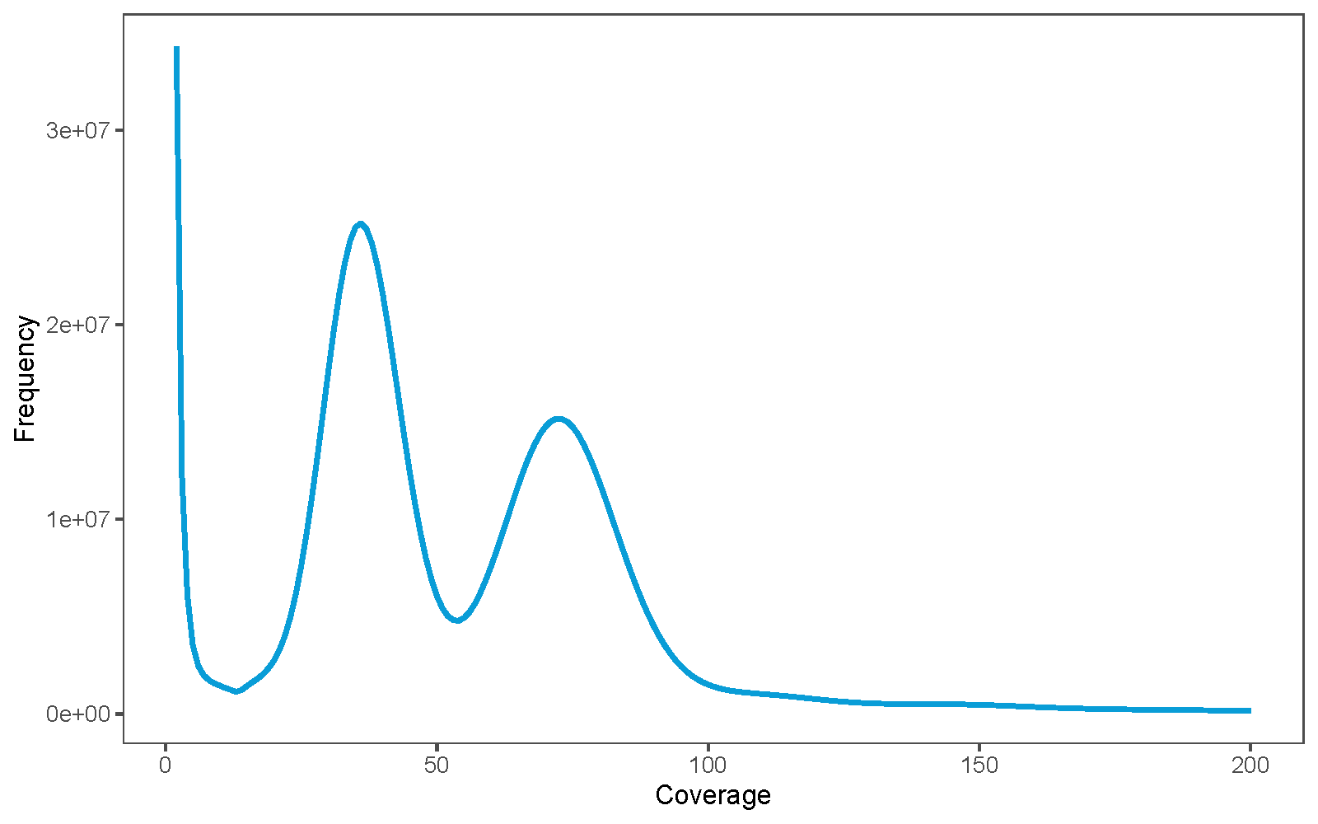
**

**Supplementary Figure S2.** 19-mers depth distribution of 308 Gb raw sequence data from libraries with insert size 300 bp. Two peaks are observed (37x and 74 x): the first peak represents frequency distribution of 17-mers from heterozygous regions and the second peak is for homozygous regions.


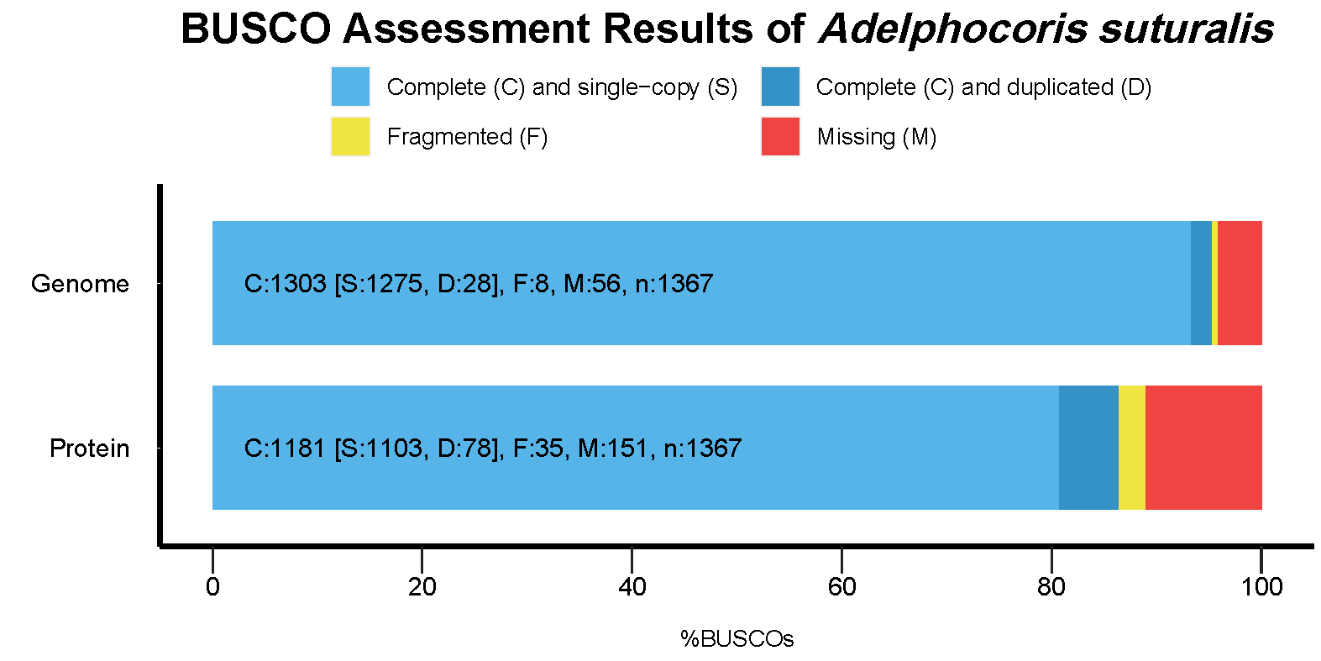


**Supplementary Figure S3.** BUSCO Assessment of *A. suturalis* genome using insecta _odb10.


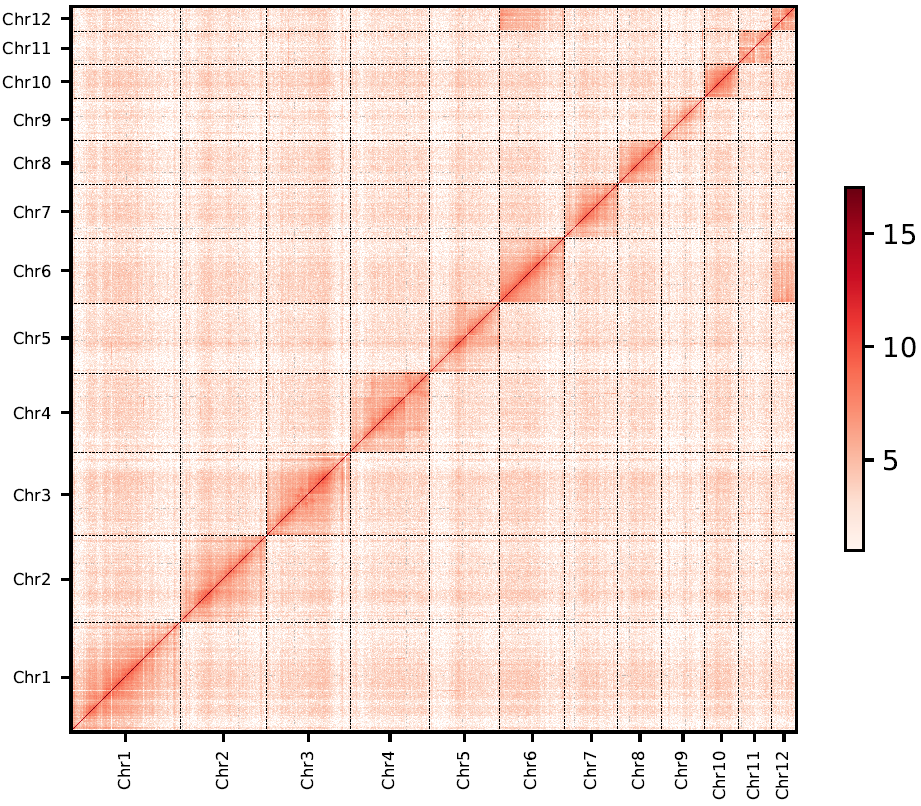


**Supplementary Figure S4.** Hi-C map of the *A. suturalis* genome showing genome-wide all-by-all interactions.


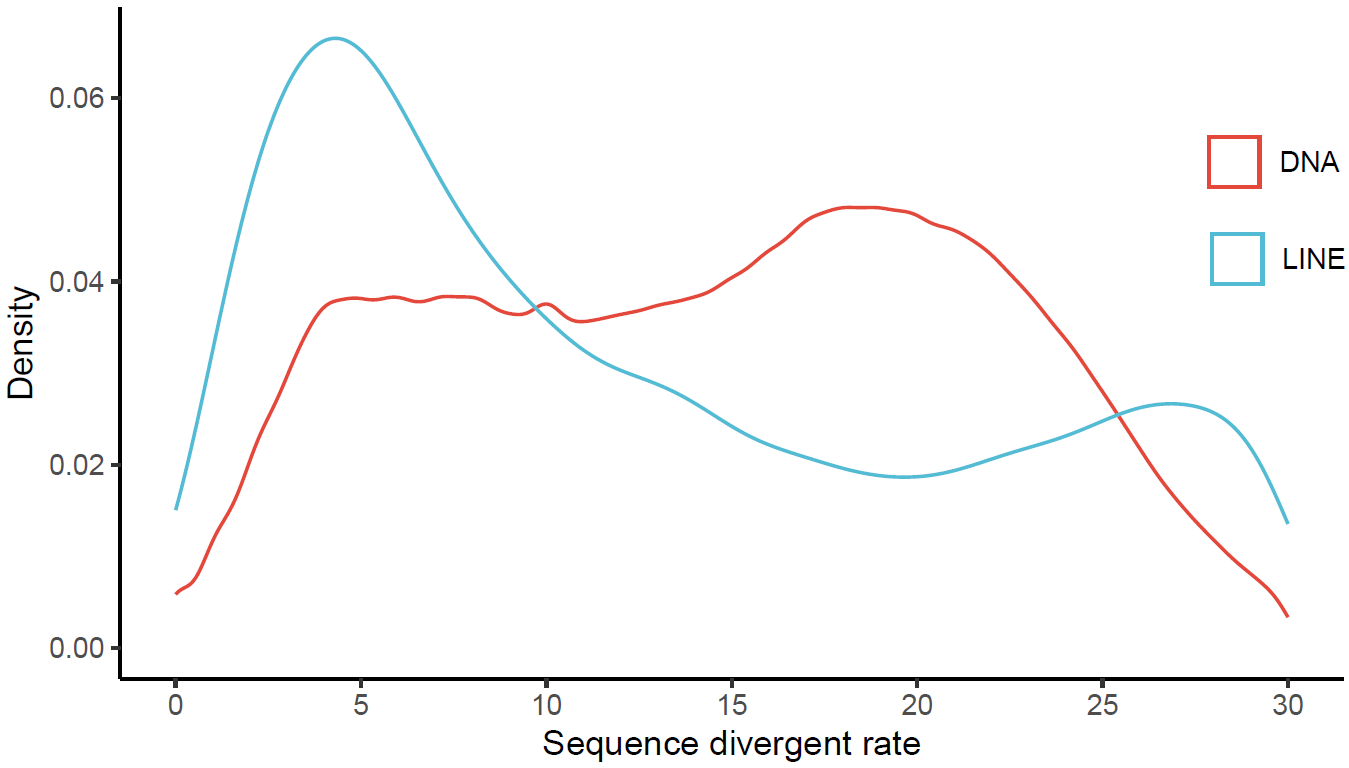


**Supplementary Figure S5.** Distribution of divergence rate of different types of TEs in the *A. suturalis* genome.

**
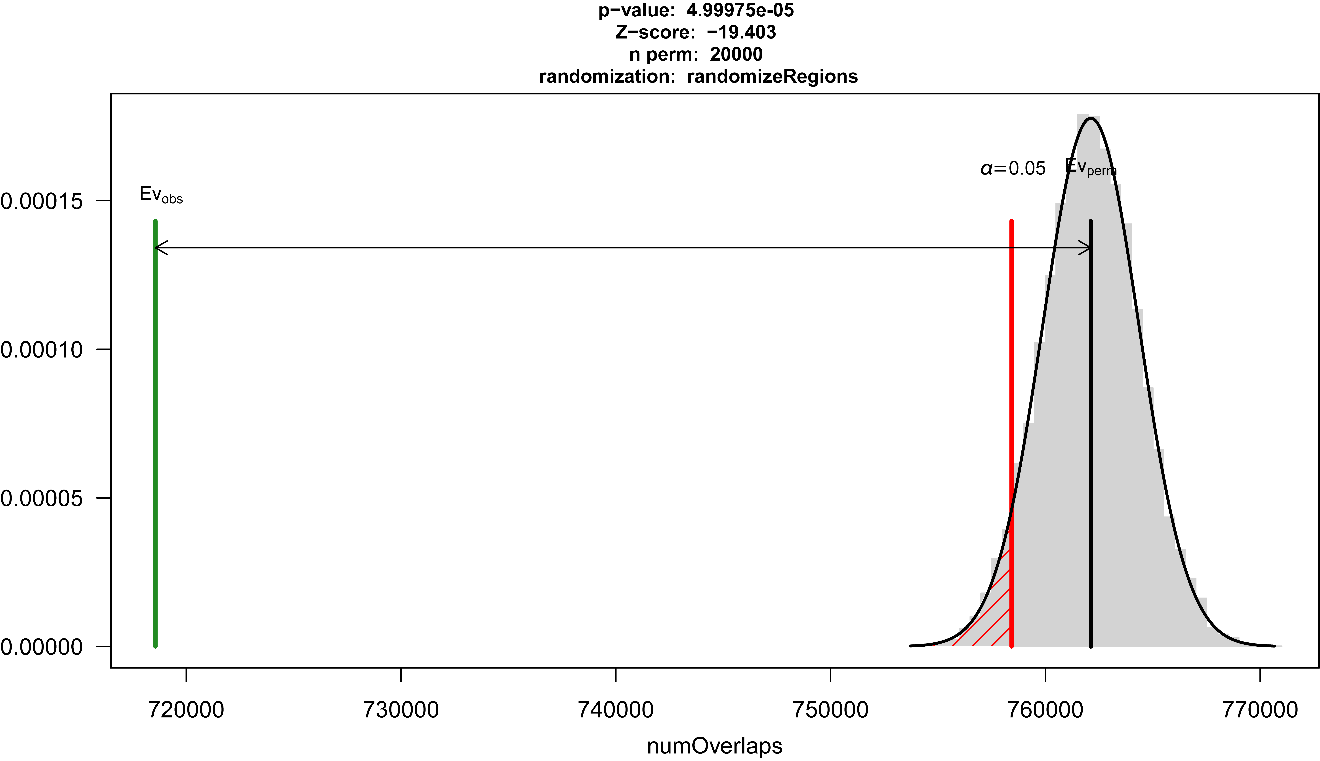
**

**Supplementary Figure S6.** Permutation test examines the correlation between the density of genes and TEs. The gray histogram representing the evaluation of the randomized regions with a fitted normal, a black bar representing the mean of the randomized evaluations and a green bar representing the evaluation of the original regions. In addition, a red bar (and red shading) represents the significance limit (by default 0.05).


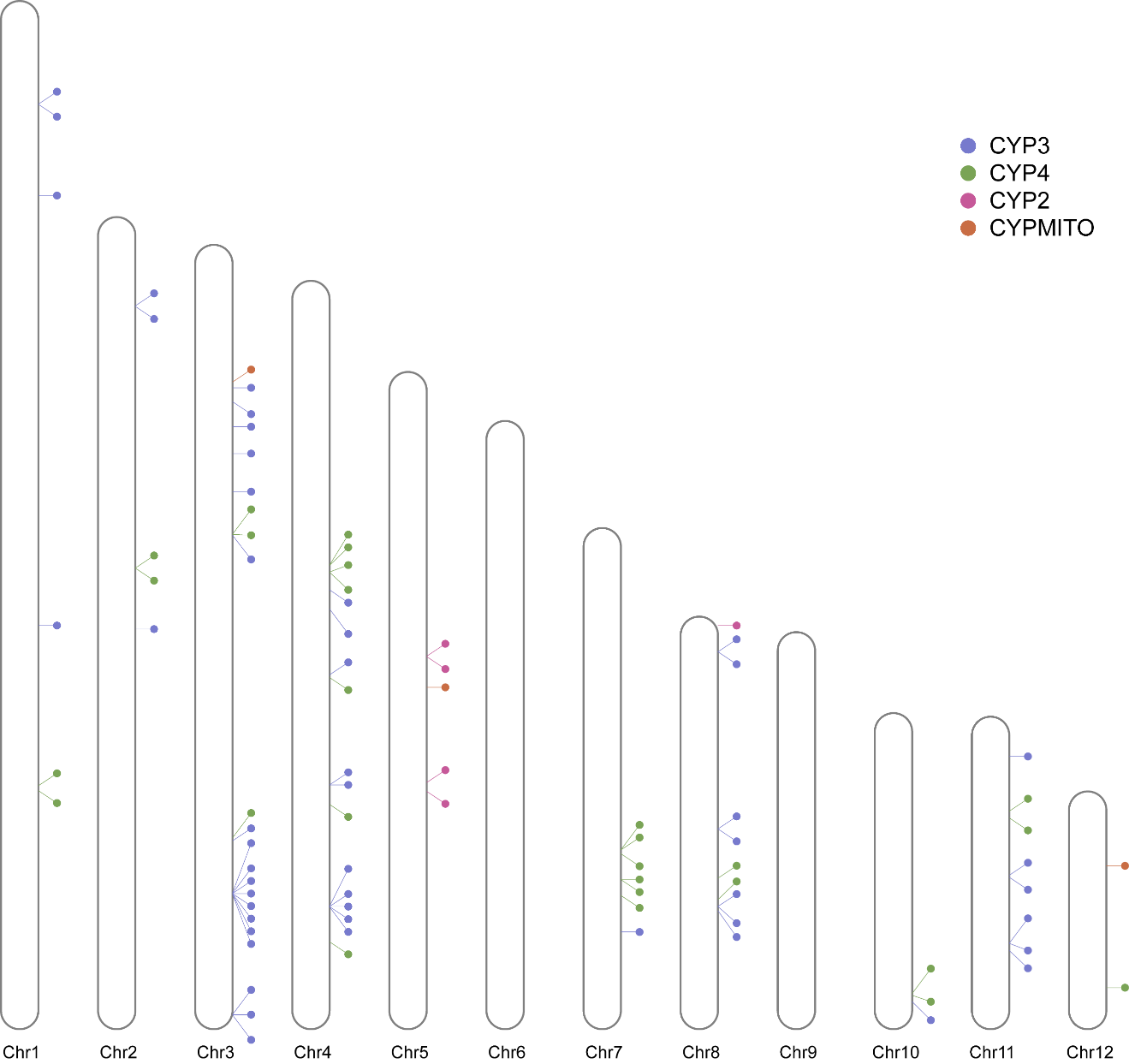


**Supplementary Figure S7.** Distribution of 86 CYP genes in the *A. suturalis* chromosomes.


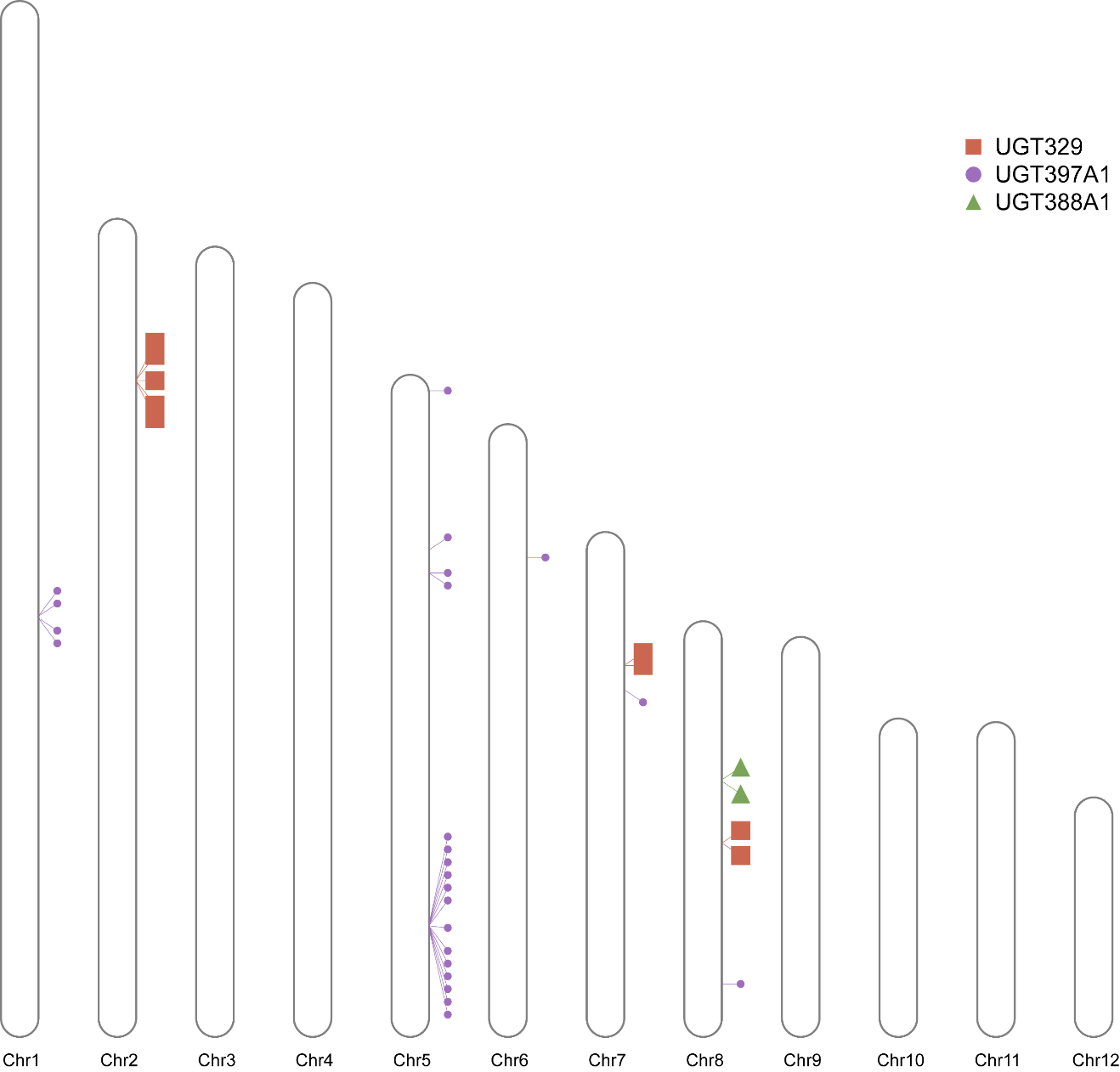


**Supplementary Figure S8.** Distribution of 35 UGT genes in the *A. suturalis* chromosomes.


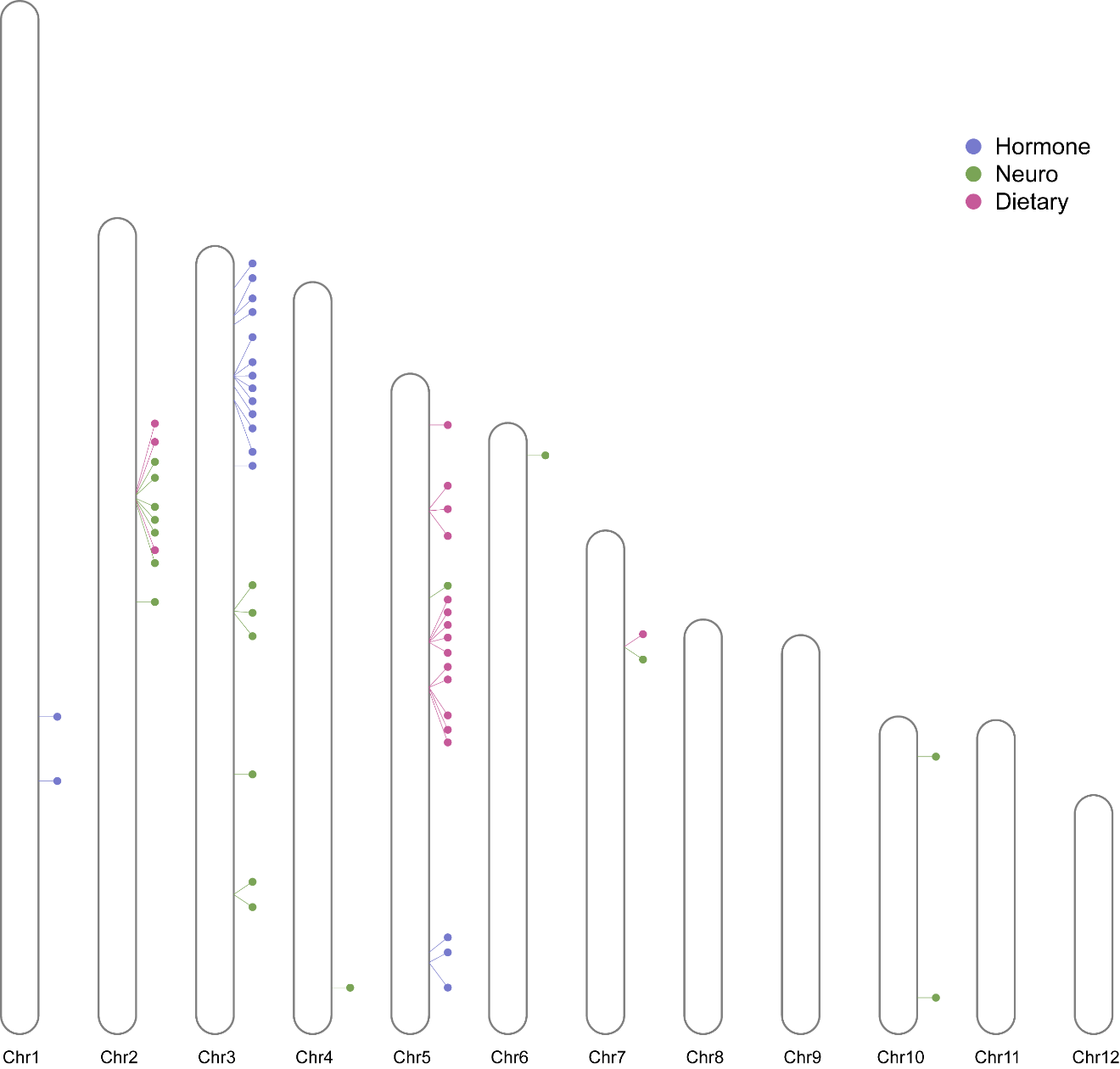


**Supplementary Figure S9.** Distribution of 55 CCE genes in the A. suturalis chromosomes.


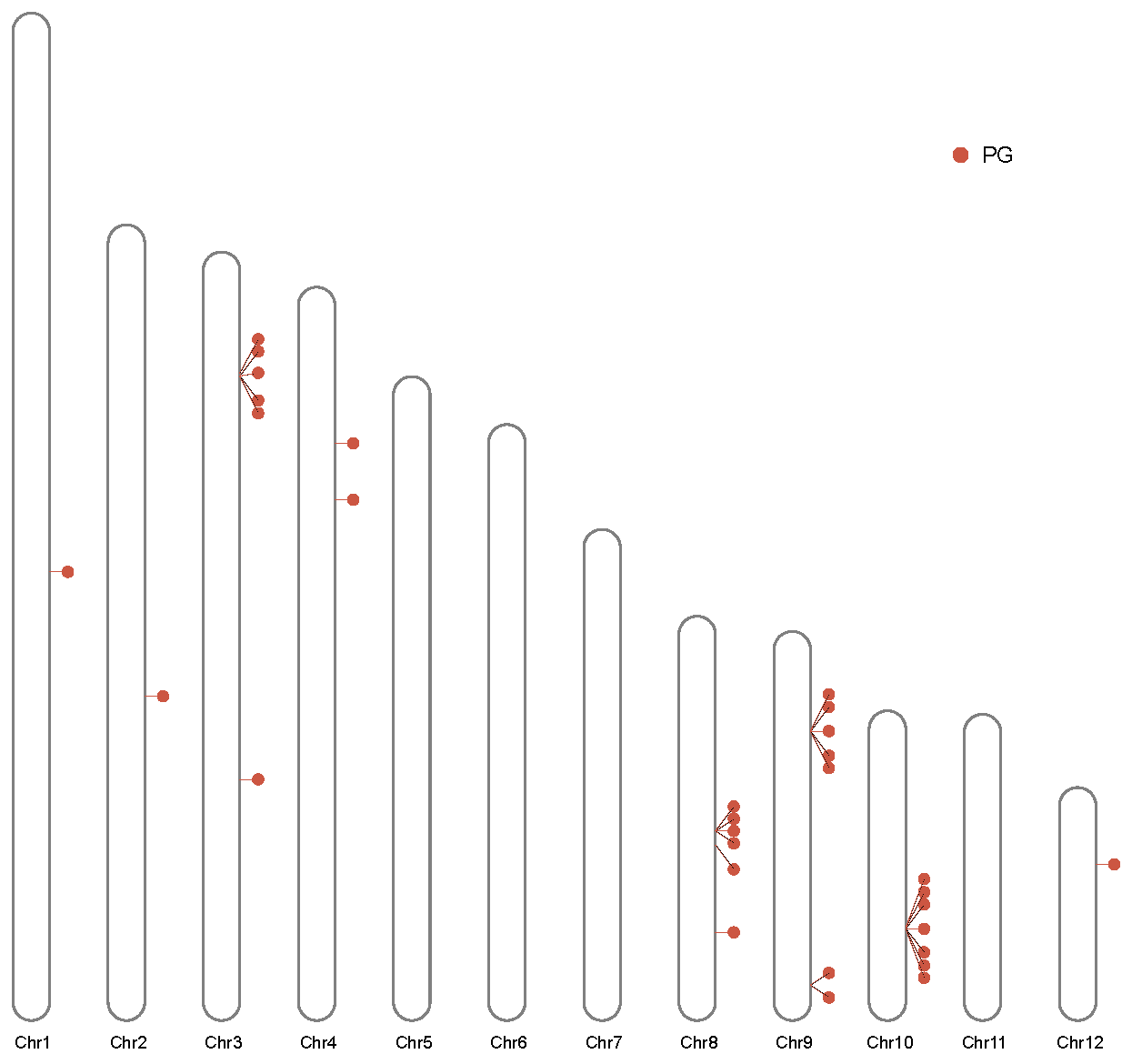


**Supplementary Figure S10.** Distribution of 31 PG genes in the A*. suturalis* chromosomes.


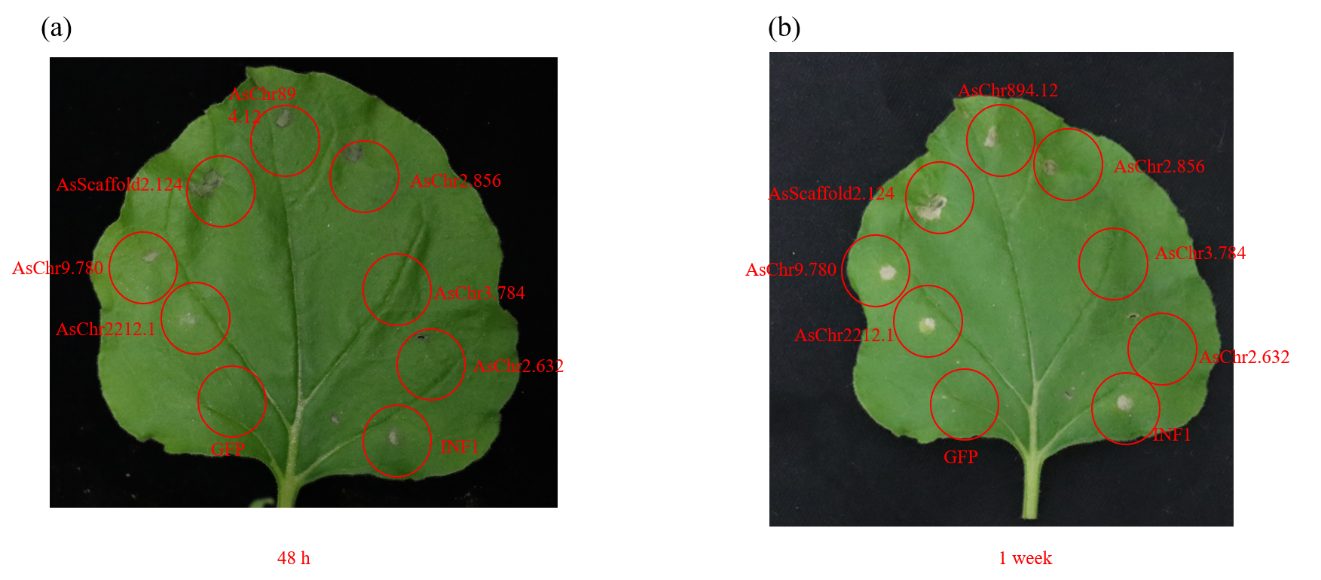


**Supplementary Figure S11.** Identification of *A. suturalis* candidate effector proteins. 48 hours **(a)** and one week **(b)** after the injection of *Agrobacterium tumefaciens*. Candidate effectors, the cell death-inducing gene *INF1*, and empty control (*GFP*) were expressed in *N. benthamiana* leaves via agroinfiltration. *GFP* is used as the negative control, and *INF1* is the positive control that induced cell death.


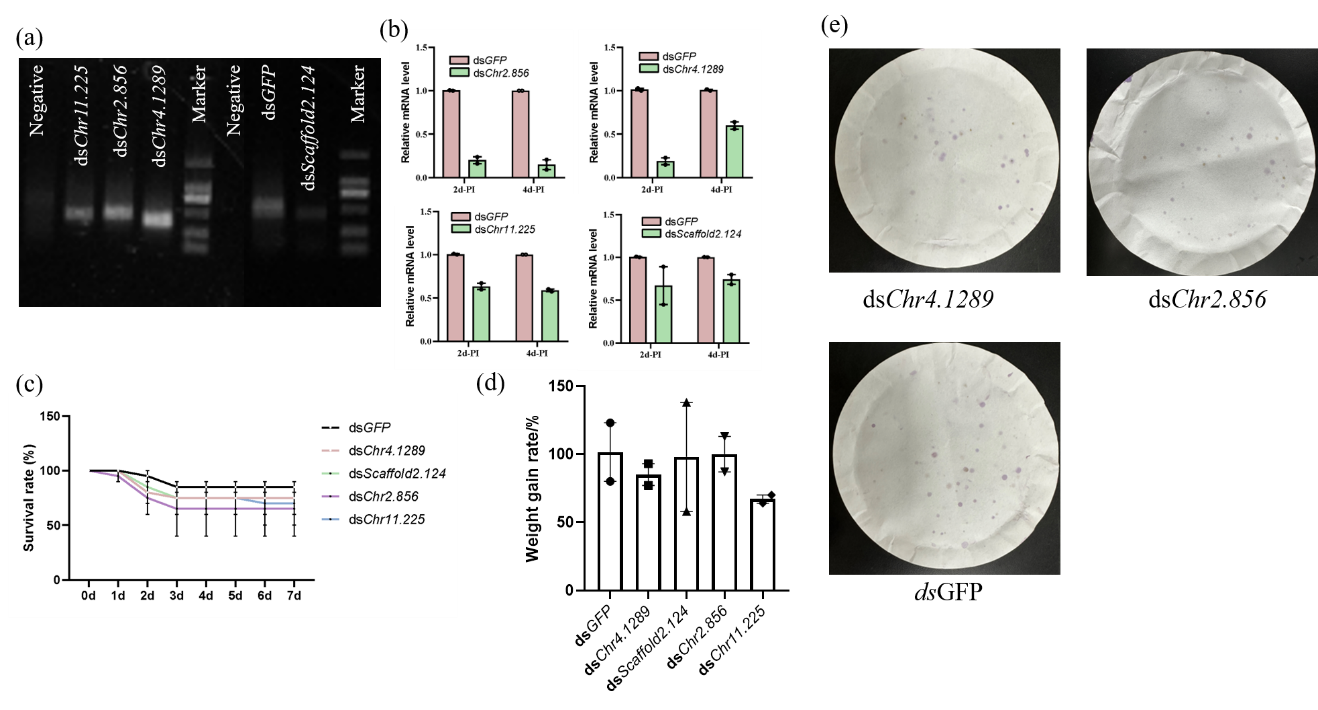


**Supplementary Figure S12.** **RNAi of *Chr2.856*, *Chr11.225*, *Scaffold2.124* and *Chr4.1289* effects on feeding behaviour for *A. suturalis*.** **(a)** PCR amplification of target fragments of dsRNA. **(b)** Mean transcript levels of *Chr2.856*, *Chr11.225*, *Scaffold2.124* and *Chr4.1289* in *A. suturalis* that had been injected with dsRNA of *Chr2.856*, *Chr11.225*, *Scaffold2.124* and *Chr4.1289* or GFP (dsGFP). Data represent mean ± SD. **(c)** Survival rate of *A. suturalis* after injection for 7 days. Data represent mean ± SD, n = 10. **(d)** The weight change curve of *A. suturalis* after ingesting dsRNA. The experiment was repeated three times. Data represent means mean ± SD. **(e)** honeydew excretion by *A. suturalis* on filter paper. The experiment was repeated three times. The green fluorescent protein (GFP) in RNAi analysis was a negative control for RNAi.

## 3. References

1. Wang H, Shi Y, Wang L, Liu S, Wu S, Yang Y, Feyereisen R, Wu Y: **CYP6AE gene cluster knockout in Helicoverpa armigera reveals role in detoxification of phytochemicals and insecticides**. *Nature Communications* 2018, **9**(1):4820.

2. Peng T, Pan Y, Gao X, Xi J, Zhang L, Ma K, Wu Y, Zhang J, Shang Q: **Reduced abundance of the CYP6CY3-targeting let-7 and miR-100 miRNAs accounts for host adaptation of Myzus persicae nicotianae**. *Insect Biochemistry and Molecular Biology* 2016, **75**:89-97.

3. Allocati N, Federici L, Masulli M, Di Ilio C: **Glutathione transferases in bacteria**. *The FEBS Journal* 2009, **276**(1):58-75.

4. Tian L, Song T, He R, Zeng Y, Xie W, Wu Q, Wang S, Zhou X, Zhang Y: **Genome-wide analysis of ATP-binding cassette (ABC) transporters in the sweetpotato whitefly, Bemisia tabaci**. *BMC Genomics* 2017, **18**(1):330.

5. Zhao Z, Zera AJ: **Differential lipid biosynthesis underlies a tradeoff between reproduction and flight capability in a wing-polymorphic cricket**. *Proceedings of the National Academy of Sciences* 2002, **99**(26):16829-16834.

6. Broehan G, Kroeger T, Lorenzen M, Merzendorfer H: **Functional analysis of the ATP-binding cassette (ABC) transporter gene family of Tribolium castaneum**. *BMC Genomics* 2013, **14**(1):6.

7. Wu C, Chakrabarty S, Jin M, Liu K, Xiao Y: **Insect ATP-Binding Cassette (ABC) Transporters: Roles in Xenobiotic Detoxification and Bt Insecticidal Activity**. *International Journal of Molecular Sciences* 2019, **20**(11):2829.

8. Jackson CJ, Liu J-W, Carr PD, Younus F, Coppin C, Meirelles T, Lethier M, Pandey G, Ollis DL, Russell RJ *et al*: **Structure and function of an insect α-carboxylesterase (*αEsterase7*) associated with insecticide resistance**. *Proceedings of the National Academy of Sciences* 2013, **110**(25):10177-10182.

9. Durand N, Carot-Sans G, Chertemps T, Montagné N, Jacquin-Joly E, Debernard S, Maïbèche-Coisne M: **A diversity of putative carboxylesterases are expressed in the antennae of the noctuid moth Spodoptera littoralis**. *Insect Molecular Biology* 2010, **19**(1):87-97.

10. Singh NK, Singh H, Singh NK, Rath SS: **Multiple mutations in the acetylcholinesterase 3 gene associated with organophosphate resistance in Rhipicephalus (Boophilus) microplus ticks from Punjab, India**. *Veterinary Parasitology* 2016, **216**:108-117.

11. Oakeshott J, Claudianos C, Campbell P, Newcomb R, Russell R: **Biochemical genetics and genomics of insect esterases**. *Comprehensive molecular insect science Volume* 2010, **5**.

12. Chen W, Hasegawa DK, Kaur N, Kliot A, Pinheiro PV, Luan J, Stensmyr MC, Zheng Y, Liu W, Sun H *et al*: **The draft genome of whitefly Bemisia tabaci MEAM1, a global crop pest, provides novel insights into virus transmission, host adaptation, and insecticide resistance**. *BMC biology* 2016, **14**(1):110-110.
